# Multiclass Cyanotoxins in Aquatic Environments: Occurrence, Impacts and Detection Techniques

**DOI:** 10.3390/toxins18090395

**Published:** 2026-09-14

**Authors:** Galina Nugumanova

**Affiliations:** Nazarbayev University Research Administration, Nazarbayev University, Astana 010000, Kazakhstan; galina.nugumanova@nu.edu.kz

**Keywords:** cyanotoxins, cyanoHABs, microcystin, nodularin, cylindrospermopsin, anatoxin-a, BMAA, saxitoxin, LC-MS/MS, multiclass analysis

## Abstract

Cyanobacterial harmful algal blooms (cyanoHABs) typically involve the release of a wide spectrum of toxic metabolites produced by different cyanobacteria, which can cause adverse health effects in animals and humans. At the same time, many cyanobacteria are capable of producing more than one toxin simultaneously. As a result, the multiclass toxins released into aquatic environments can exert complex and combined effects on various ecosystem components. This review aims to provide an overview of the occurrence and effects of different cyanotoxins in aquatic systems and current analytical methods of their simultaneous detection. The main classes of cyanotoxins have been considered in this review. The occurrences, properties, and environmental and health impacts of these toxins are described. The main methods for cyanobacterial toxin analysis are covered. A comprehensive review and analysis of liquid chromatography-tandem mass spectrometry (LC-MS/MS) detection and sample preparation approaches used to identify various classes of cyanotoxins is provided, together with a practical decision tree for analytical workflow selection according to toxin class and sample matrix, highlighting key trends and outlining directions for further research in this area.

## 1. Introduction

Cyanobacteria are among the most ancient and adaptable microorganisms on Earth, capable of thriving in a wide range of environments [1]. Cyanobacteria are capable of nitrogen fixation and also play an important role in carbon cycling by assimilating carbon dioxide through photosynthesis into organic compounds and oxygen [2,3]. Under favorable conditions, these prokaryotic organisms tend to grow vigorously, forming cyanobacterial harmful algal blooms (cyanoHABs). CyanoHABs are now occurring more often, lasting for longer periods, and covering broader regions worldwide [4,5]. The increase in anthropogenic impact on the environment in combination with climate fluctuations leads to an increase in cyanoHABs and, as a consequence, to an increase in the risks of harmful effects on the environment, animals and humans, expressed primarily in poisoning by cyanotoxins released by some types of cyanobacteria [3,4,5].

Acute poisoning events have been reported in various regions worldwide [6,7,8]. First, these are massive fish kills that may be linked to cyanotoxin poisoning. Such cases have been reported, for example, in Algeria, where fish deaths were suspected to be linked to the toxic cyanobacterial *Planktothrix* bloom in the Béni-Haroun Reservoir [9]. Cyanobacterial bloom of *Aphanizomenon* was associated with a massive fish kill in Lake Vistonis (Greece) [10]. A large number of cases are associated with the poisoning of oysters, mussels, and other shellfish due to their filter feeding, which implies consuming large volumes of water accumulating toxins produced by cyanobacteria [11,12]. Cyanotoxin intoxication can affect birds [13,14], livestock [15], and wild and domestic animals [16,17,18,19], including megafauna [20]. Cyanotoxins have also been detected in agricultural crops [21] and other plants [22] due to contamination during irrigation. Cyanotoxin poisoning also occurs in humans [23]. It is worth noting that some cyanobacteria can release different cyanotoxins simultaneously, and the same type of cyanotoxin can be produced by different cyanobacterial genera [24,25,26]. As a result, a complex mixture of cyanotoxins can be produced during a single cyanoHAB event.

The abovementioned and many other cases of cyanotoxin poisoning demonstrate the importance of monitoring and regulating cyanotoxins in food, water, and other environmental sources. Thus, the World Health Organization (WHO) has established guidance values concerning several cyanotoxins for drinking water and recreational activities, as they have adverse health effects [27,28,29]. At the same time, existing guidelines only cover the most common cyanotoxins, and, therefore, the development of methods for detecting a wide range of multiclass cyanotoxins remains relevant to this day [30]. Numerous methods exist for determining the toxicity of cyanobacteria and the toxins themselves, including molecular methods, biochemical assays, and physicochemical methods, with liquid chromatography coupled with mass spectrometric detection (LC-MS) being the main physicochemical technique used for quantitative analysis of cyanotoxins [30].

The aim of this study is to highlight the existing knowledge about multiclass cyanotoxins in aquatic environments and systematize approaches to the extraction and detection of multiclass cyanotoxins. Previous reviews [30,31,32,33] mainly focused on cyanotoxin occurrence, classification, risk assessment and general cyanotoxin detection methods. Although some of these reviews briefly mention the possibility of simultaneous determination of multiple cyanotoxins, the corresponding analytical strategies are not comprehensively discussed. In contrast, alongside an overview of cyanotoxins, this work places particular focus on the comparative analysis of analytical and sample preparation strategies, as well as workflow selection, for the simultaneous determination of multiclass cyanotoxins in different sample matrices, with an emphasis on liquid chromatography-tandem mass spectrometry (LC-MS/MS) methods.

The literature search for this review was primarily conducted through Google Scholar and Web of Science and focused on publications from the past 15 years (2010–2025). Peer-reviewed journal articles were the main source of information, supplemented by relevant books, book chapters, technical reports, and other authoritative sources. Additional relevant references cited in the identified publications were included where appropriate. Dissertations and theses were excluded. Different combinations of keywords were used depending on the topic addressed in each section of the review, including “cyanotoxins”, “multiclass cyanotoxins”, “cyanotoxin detection”, and “simultaneous cyanotoxin analysis”. Only English-language sources were considered. Publications were selected based on their relevance to the scope of the review and the specific topics addressed in each section.

## 2. Cyanotoxins and Aquatic Environments

Due to climate change, eutrophication, and the expansion of urban settlements without adequate wastewater treatment, cyanobacterial blooms continue to intensify, posing health risks. There are numerous comprehensive reviews dedicated to the study of cyanotoxins, their environmental distribution, and ecological significance [8,24,34,35,36]. Because cyanobacteria are able to tolerate large variations in salinity, temperature, and light intensity, their ability to survive in diverse environments is high, giving them a significant competitive advantage over other microorganisms [8,34]. This also expands their range of distribution to areas with different climatic characteristics, exposing the corresponding aquatic environments and surrounding areas to the possibility of blooms and pollution with toxic metabolites [8,34,36].

### 2.1. Cyanobacterial Harmful Algal Blooms

In recent years, cyanoHABs have become a serious global problem, with cases recorded worldwide under varying climatic and hydrological conditions [31,37,38]. Elevated nutrient inputs from agriculture intensification, increase and development of recreational areas, expansion of fish farming activities, and global warming together create favorable conditions for cyanobacterial growth and toxin production [4,39,40]. The number of studies on the detection of various toxic metabolites of cyanobacteria is expanding. Cyanotoxins have been reported in the USA [40], Brazil [41], China [42], Canada [43], South Africa [44], Russia [45], UK [46], Vietnam [47], etc. Recent studies have revealed the presence of microcystins (MCs) in the polar regions of the Arctic and Antarctic [37], and cyanotoxins have also been detected in the desert [48].

In addition, water from blooming sources can be carried by currents over very long distances, transporting toxins and endangering areas remote from the epicenter of the bloom. For example, in Upper Klamath Lake (Oregon, USA), a bloom of the nitrogen-fixing cyanobacterium *Aphanizomenon flos-aquae* was found to carry polluted water downstream along the Klamath River, reaching two large stratified reservoirs, Copco and Iron Gate, the latter of which was identified as the primary source of *Microcystis* in downstream river sections [49]. Therefore, cyanoHABs containing MCs were transported 300 km downstream into the Pacific Ocean, degrading fisheries and water quality along the way [49,50]. There are numerous studies addressing the transfer of cyanotoxins from freshwater bodies to marine environments, including investigations into the mechanisms of their release, as well as the coexistence of multiple toxin classes, both cyanobacterial and algal toxins, such as domoic acid (DA) [51,52,53,54].

Exposure to cyanobacterial toxins may alter wildlife diversity and abundance. Toxins may accumulate in aquatic organisms and spread through the food chain, affecting other species [55]. CyanoHABs degrade water quality, disrupt ecosystem functioning, and pose a threat to fisheries, aquaculture, and public health. This highlights the critical role of effective detection and monitoring methods to ensure water quality and protect ecosystems and human health from the growing threat of cyanoHABs and cyanotoxin contamination.

### 2.2. Diversity of Cyanobacterial Toxins

**Figure 1:**
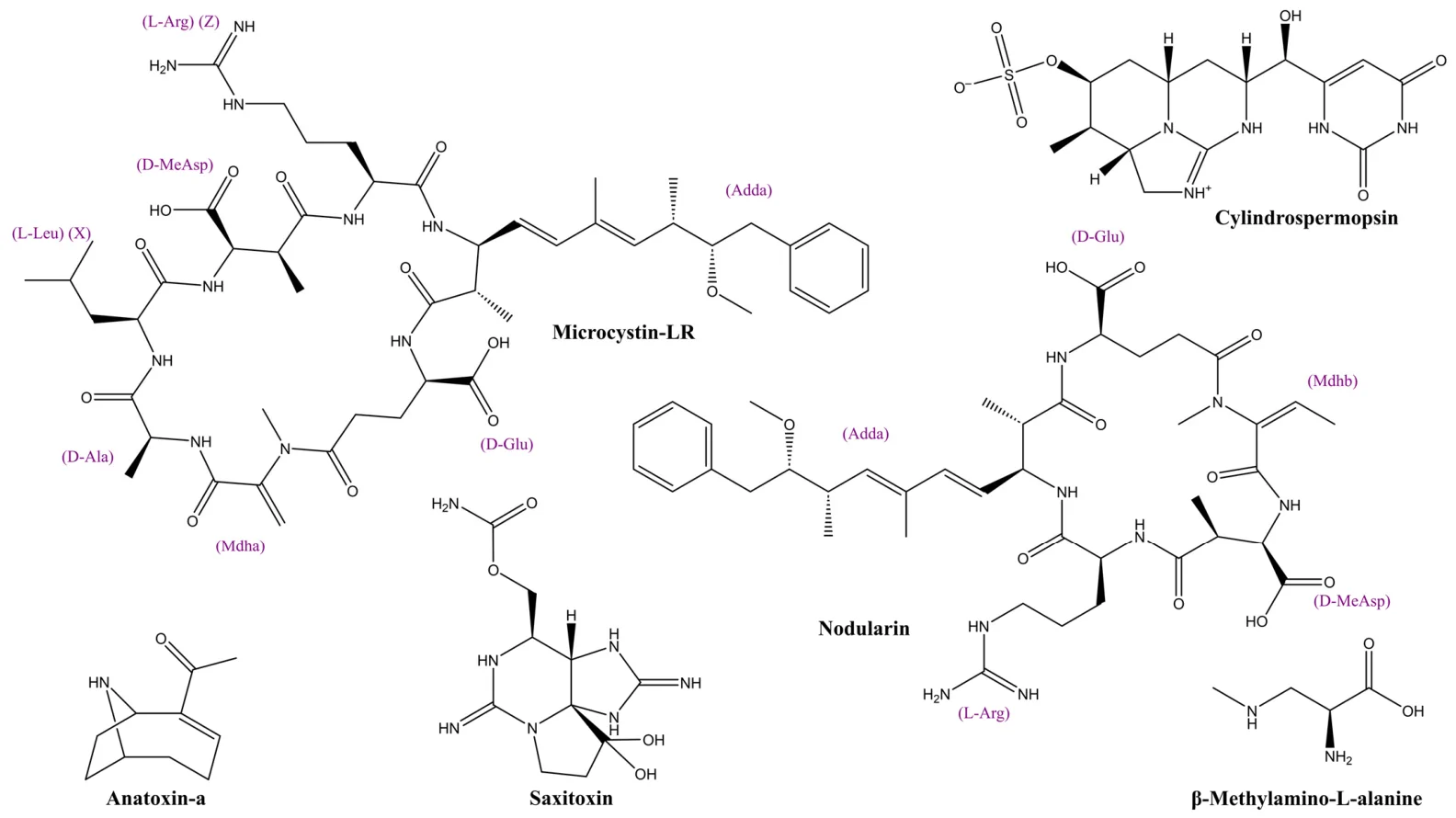
Chemical structures of selected cyanobacterial toxins.

Cyanobacteria produce a wide range of toxins with diverse chemical structures and modes of action. The release of cyanotoxins into the environment may occur during cell death or without cell rupture [1]. Based on functional properties, toxins can be categorized into the following groups: hepatotoxins, neurotoxins, dermatotoxins, and cytotoxins [26]. Lipopolysaccharides (LPSs), classified as endotoxins, are components of the cyanobacterial outer membrane and can exhibit toxic properties [1,31]. They can also be divided into classes based on their physicochemical properties. Cyclic peptides are represented by the microcystin family and nodularins (NODs). The alkaloids include anatoxin-a (ATX-a), guanitoxin (GNT, also known as anatoxin-a(S)), cylindrospermopsin (CYN), saxitoxins (STXs), aplysiatoxins and lyngbyatoxin [35]. Another class of cyanotoxins comprises non-proteinogenic amino acids related to neurotoxins, including β-methylamino-L-alanine (BMAA) with isomers 2,4-diaminobutyric acid (2,4-DAB) and *N*-(2-aminoethyl)glycine (AEG) [48]. The major cyanobacterial toxins of each class are shown in Figure 1.

MCs are the most frequently reported toxins in cyanoHABs in human and animal poisonings [56]. MCs are primarily produced by freshwater cyanobacteria of the genera *Microcystis*, *Dolichospermum* (formerly planktonic *Anabaena*), *Nostoc*, *Planktothrix* and *Anabaenopsis* [25]. The structure of microcystins is cyclo-(D-alanine-X-D-MeAsp-Z-Adda-D-glutamate-Mdha) in which X and Z are variable L-amino acids, D-MeAsp is D-*erythro*-β-methylaspartic acid, and Mdha is N-methyldehydroalanine. Adda ((2*S*,3*S*,4*E*,6*E*,8*S*,9*S*)-3-amino-9-methoxy-2,6,8-trimethyl-10-phenyldeca-4,6-dienoic acid) is a unique non-proteinogenic amino acid characteristic of cyanobacterial peptides such as MCs and NODs [1,35]. More than 270 different variants of MCs have been identified to date [57,58]. The most abundant and studied variant of MCs is microcystin-LR (MC-LR). Microcystin-RR (MC-RR) and microcystin-YR (MC-YR) are common analogues most frequently identified along with MC-LR [57]. Most of the common MCs (such as MC-LR and MC-RR) are water-soluble and remain stable under natural sunlight, resistant to hydrolysis, oxidation at neutral pH, and boiling [35]. These toxins are degraded by microorganisms in surrounding waters [59]. Oxidation of MCs can occur in the presence of ozone or other strong oxidizing agents [35].

The NODs are cyclic pentapeptides structurally similar to MCs, containing D-MeAsp, L-arginine, Adda, D-glutamate and N-methylated dehydrobutyrine residue α-(methylamino)-dehydrobutyric acid (Mdhb) and are also a widespread and commonly occurring cyanotoxin group. NODs are mainly produced by *Nodularia spumigena*, but recently another producer has been identified—*Iningainema pulvinus* (Scytonemataceae) [60]. There are 10 congeners of nodularin, with nodularin-R (NOD-R) being the most common and widely investigated [31,61]. Like microcystins, NODs are water soluble and relatively chemically stable compounds and can also be degraded by microorganisms [62,63,64]. They show resistance to temperature, light, and microwaves [61,63].

Cylindrospermopsins (CYNs) are alkaloids comprising a tricyclic guanidino moiety linked via an hydroxylated bridging carbon (C7) to uracil. To date, five structural variants of CYN have been identified [1,31]. Many cyanobacterial genera can produce CYN: *Raphidiopsis* (formerly *Cylindrospermopsis*), *Aphanizomenon*, *Umezakia*, *Chrysosporum*, and *Dolichospermum*. CYN is stable across a wide range of temperatures and pH values, with degradation occurring at high temperatures and alkaline conditions [1,34,65]. CYN may undergo microbial degradation in natural environments [66]. It remains relatively stable in both dark and light [1].

Anatoxins (ATXs) are potent neurotoxins. The most often identified is ATX-a. ATX-a is an alkaloid, a secondary amine, 2-acetyl-9-azabicyclo(4-2-1)non-2-ene. Homoanatoxin-a (hATX) is an anatoxin-a homologue, which, unlike ATX-a, has a propionyl group at C-2 instead of the acetyl group in the ATX-a structure [24]. ATX-a is produced by many cyanobacteria, including *Aphanizomenon*, *Cuspidothrix*, *Raphidiopsis*, *Cylindrospermum*, *Dolichospermum*, *Microcystis*, *Oscillatoria*, *Planktothrix*, *Tychonema*, *Phormidium*, and *Kamptonema* [1,24,67]. ATX-a is highly soluble in water and relatively stable in the dark, but it degrades rapidly in sunlight, especially at higher pH [67]. Biodegradation of ATX-a by microorganisms has also been reported [68].

STXs are a group of carbamate alkaloid neurotoxins referred to as the paralytic shellfish poisoning (PSP) toxins. These toxins are either nonsulfated (STXs), monosulfated (gonyautoxins (GTXs)), or disulfated (C-toxins) [24]. The chemical structure consists of a tetrahydropurine group and two guanidine subunits [29]. More than 50 STX analogues have been described [69]. STXs are produced by marine dinoflagellates and by freshwater cyanobacteria, including genera *Aphanizomenon*, *Dolichospermum*, *Microseira* (previously *Lyngbya*), *Planktothrix* and *Raphidiopsis* [11,29]. STXs are highly soluble in water and stable at high temperatures and under acidic conditions [70]. STXs can be degraded in natural environments by bacterioplankton and sunlight [71].

BMAA is a non-proteinogenic amino acid with neurotoxic properties, which can be produced by multiple groups of diatoms, dinoflagellates, both symbiotic and free-living cyanobacteria [72,73]. BMAA and its congeners (AEG and 2,4-DAB) have been found to be produced by 29 different genera of cyanobacteria, including *Nostoc*, *Dolichospermum*, *Raphidiopsis*, *Leptolyngbya*, *Nodularia*, *Planktothrix*, and *Synechocystis* [74]. These kinds of toxins are most persistent under cold, dark conditions and degrade rapidly when exposed to natural sunlight and higher temperatures, especially in the presence of organic matter in the water [75].

Many toxin-producing genera are capable of synthesizing more than one toxin [1,74,76,77,78,79]. Moreover, toxin production can differ among strains of the same species [1,80]. According to Sivonen [24], some strains of *Aphanizomenon flos-aquae* and *Dolichospermum circinale* (formerly *Anabaena circinalis*) can simultaneously produce several neurotoxins—STX, gonyautoxin 1 (GTX1), gonyautoxin 3 (GTX3), and gonyautoxin 4 (GTX4). It has been shown that the cyanobacterium *Aetokthonos hydrillicola*, which produces the recently discovered aetokthonotoxin (AETX), also produces other cyanotoxins known as aetokthonostatins [81,82]. Individual strains of *Microcystis aeruginosa* can produce different MC congeners at varying concentrations [57]. At the same time, Pitois et al. [83], while determining correlations between the biomasses of the detected cyanobacterial species and the quantitatively detected toxins, having determined distribution patterns for some microcystins, suggested the possible production of MC-LR by almost all potentially toxic cyanobacterial species observed in the study samples [83]. Table 1 presents cyanobacterial genera, certain species of which may be capable of producing various cyanotoxins.

Within the reviewed genera, MCs are the most widely distributed cyanotoxin class apart from BMAA, occurring in various combinations with other cyanotoxins. *Microcoleus* has been reported as a producer of ATX-a [1]. Recently one strain of *Microcoleus autumnalis* has been found to contain a fragment of the *mcyE* gene. Enzyme-linked immunosorbent assays (ELISAs) confirmed that culture extracts contained microcystin [78]. The microcystin-producing *Raphidiopsis raciborskii* strain has also been reported [87]. *Dolichospermum circinale* is a known producer of a complex of STXs, including STX, monosulfated (gonyautoxin 2 (GTX2) and GTX3) and disulfated (N-sulfocarbamoylgonyautoxin-2 (C1) and N-sulfocarbamoylgonyautoxin-3 (C2)) toxins [110]. *Kamptonema* sp. PCC 6506 (formerly *Oscillatoria* sp. PCC 6506) has been reported to produce ATXs and CYNs [84,85].

*Microcystis* is primarily associated with microcystin production; however, BMAA production has also been reported in two *Microcystis* strains, PCC 7806 and PCC 7820 [73]. Furthermore, Park et al. [111] demonstrated for the first time that *Microcystis* strains TAC 87 and TAC 117 simultaneously produced ATX-a and MCs. ATX-a production by *Cylindrospermum* was reported by Sivonen et al. [112]. Later, the *Cylindrospermum stagnale* strain BC 144 was found to produce several STXs [79].

The production of neurotoxins and hepatotoxins is characteristic of the genus *Planktothrix*, which is able to produce STXs, ATX-a, BMAA and MCs [31,67,74,113,114]. *Raphidiopsis* is capable of producing of MCs, CYNs, ATX-a, STXs, and BMAA [1,67,74,87,115]. *Dolichospermum flos-aquae* strain NRC 525-17 produces GNT and several MCs, indicating the simultaneous production of multiclass cyanotoxins [116,117]. However, numerous questions remain regarding the mechanisms and patterns of toxin production by different species under varying environmental conditions require further investigation to be elucidated.

### 2.3. Bioaccumulation of Cyanotoxins and Their Impacts on Aquatic Food Webs

Cyanotoxins can accumulate at different levels of the trophic chain [118,119]. Considering the biomagnification of freshwater cyanotoxins, it should be noted that the distribution and accumulation of toxins vary across trophic levels [119]. By consuming phytoplankton enriched in cyanotoxin-producing species, some animals, such as bivalves, accumulate greater amounts of toxins than fish, which may be due to the latter’s more efficient detoxification mechanisms [119]. Most of the research relates to the study of the distribution of the MCs. Reported concentrations reach up to 38.48 μg g^−1^ dry weight (dw) in mollusks [120], 4.29 μg g^−1^ dw in freshwater shrimps, 0.93 μg g^−1^ dw in crayfish [121], and 1.5 μg g^−1^ wet weight (ww) in various fish species [122,123], including those consumed by humans. Although the concentrations of accumulated toxins are not very high, regular consumption of aquaculture products with relatively low MC levels can cause negative long-term effects on humans [124]. Low MC concentrations detected in fish from water bodies with regular cyanoHABs may be due to detoxification by aquatic inhabitants, but even so, MC exposure can negatively affect fish embryo development and behavior [124]. In the case of CYN, as with MCs, biodilution likely occurs at higher trophic levels [125]. Variation in the uptake and detoxification of the different cyanotoxins depends on the species and the toxin, including differences among toxins of the same class. The distribution of toxins in various tissues and organs of animals also differs [119,126]. NODs can accumulate in a wide range of organisms primarily in the liver [63]. Nodularin concentrations of up to 2.2 μg g^−1^ have been reported in fish liver tissues [127]. Bioaccumulation of CYNs is observed in invertebrates, for example, CYN concentrations of up to 2.52 μg g^−1^ dw have been reported in mussels [128] and up to 4.3 μg g^−1^ dw in crayfish [129]; vertebrates such as different species of fish, some mammals, targeting liver and kidneys [125]; and also in plants [1,130]. Saxitoxin (STX) causing PSP in people who consume contaminated seafood accumulates primarily in shellfish (mussels, oysters, clams, and scallops), as well as in crustaceans, fish, and marine mammals [69]. Concentrations of up to 3.67 µg STX eq. g^−1^ tissue have been reported in shellfish [131]. The location of tissues for accumulations of STXs can also vary among the species, depending on differential acquisition of toxins by individuals and feeding habits [132].

Cyanotoxin exposure, occurring directly or through trophic transfer in aquatic food webs, can pose risks to specific populations [126]. Long-term dietary exposure to cyanotoxins can lead to mortality or a range of adverse effects in animals across all trophic levels. These effects may inflict damage to vital organs and the immune system, alter behavior, and reduce growth and reproductive performance [126]. Cyanotoxins can influence the structure of the zooplankton community [133]. It was confirmed that long-term exposure to MCs was fatal to *Daphnia magna* [134]. The effects of CYN on *Daphnia magna* were also investigated, revealing abnormal swimming patterns, decreased acetylcholinesterase (AChE) activity, and reduced filtration and ingestion rates due to effects on the heart and thoracic limbs [135]. The inhibitory effect of MC-LR on photosynthesis and growth of cyanobacteria *Nostoc muscorum* and *Anabaena* BT1 has been shown [136]. STXs caused severe reductions in spontaneous swimming behavior and touch response in larval herring (*Clupea harengus pallasi*) [137]. ATX-a has been shown to influence fish behavior, as well as reproductive and predator–prey interactions, in *Cyprinus carpio*, potentially leading to negative consequences for fish populations [138].

Although numerous studies have documented the presence and accumulation of cyanotoxins at various trophic levels [7,139], understanding their accumulation mechanisms remains limited, as the biomagnification pathways of cyanotoxins across classes may differ. Nevertheless, understanding that cyanotoxins can contribute to the development of subacute and chronic effects in humans indicates the reality of the risk associated with dietary exposure. These effects range from carcinogenesis to neurodegeneration, including amyotrophic lateral sclerosis (ALS), Parkinson’s and Alzheimer’s diseases [119,140,141].

## 3. Health Impacts of Cyanotoxins

Upon uptake by animals and humans, cyanotoxins have been associated with a range of adverse health effects. These effects can range from acute physiological disruptions to long-term impacts on organ function and overall health [1]. Cyanotoxin effects vary widely depending on the class of toxin, concentration, and exposure duration. Exposure to cyanotoxins is known to cause hepatic, gastrointestinal, neurological, and carcinogenic effects [1]. The main exposure routes to cyanotoxins for humans are the consumption of contaminated drinking water that has not been effectively treated, inhalation of aerosolized blooms and skin contact during recreational water use and the consumption of food in which toxins may have accumulated (fish, shellfish, and plants) [142,143,144].

Hepatotoxins are primarily represented by MCs and NODs, which affect the metabolic homeostasis of the liver. MCs and NODs inhibit protein phosphatases 1 (PP1) and 2A (PP2A) in liver cells. Poisoning with these cyanotoxins, depending on the duration of exposure and the concentration of toxins, can have consequences of varying severity—inflammation, liver fibrosis, and liver failure [124]. Beyond their well-known hepatotoxic effects, MCs have also been shown to induce nephrotoxicity, neurotoxicity, reproductive toxicity, and endocrine disruption [145,146,147,148]. Documented cases of acute intoxication include dog fatalities associated with the direct ingestion of water contaminated by MC-producing *Microcystis aeruginosa* blooms [149] and fatal intoxication of southern sea otters linked to MC exposure [16]. One of the most significant human-poisoning events associated with MCs occurred in Caruaru (Brazil) in 1996, resulting in more than 50 deaths among hemodialysis patients [150]. Exposure to NODs may result in adverse health effects in animals and humans, including carcinogenic outcomes. Although nodularins cannot currently be classified as carcinogens due to insufficient exposure data, it has been suggested that they may be more toxic than MCs [63].

CYNs represent a class of cyanotoxins with pronounced cytotoxic effect [130,151]. CYNs have been shown to have hepatotoxic properties [152]. CYN is a potent inhibitor of protein synthesis [28]. Acute toxic events associated with CYN have been reported in cattle in Australia [153]. Recent studies have found that CYNs also cause nervous system damage and are more toxic than MCs in terms of neurotoxicity [31]. In addition to inducing cytotoxicity, CYN has been associated with skin toxicity and genotoxic effects. It has also been reported to impact kidneys (causing tubular necrosis), thymus (causing atrophy), and heart (causing pericardial and myocardial hemorrhage) [151,154]. Hercog et al. [155] reported that CYN exerts higher genotoxic potential than MC-LR and that genotoxic potential of the MC-LR/CYN mixture is comparable to that of CYN alone.

Dermatotoxic effects are mainly attributed to cyanobacterial LPS [156]. However, some other cyanotoxins are also capable of exhibiting similar properties, for example, CYN [130], aplysiatoxins and lyngbyatoxin [35].

Neurotoxins include ATXs, GNTs, STXs, BMAA and its congeners. ATXs may exert adverse effects on animal and human health. ATX-a is a nicotinic acetylcholine receptor agonist [157]. Acute toxic events associated with ATX-a have been reported in dogs, including six fatal poisonings [158]. GNT is an organophosphate with neurotoxic properties similar to those of organophosphate insecticides—parathion and malathion [25]. GNT irreversibly inhibits acetylcholinesterase, which can result in muscle overstimulation, seizures, and respiratory failure [159]. Bird mortalities caused by GNT-producing *Dolichospermum lemmermannii* has been reported [160]. STXs block voltage-gated sodium channels [11]. In marine environments, these toxins accumulate in seafood and cause PSP, affecting the nervous system [11]. STX is one of the most potent cyanotoxins (LD50 i.p. in mice 10 µg kg^−1^) [24]. Human exposure to STXs through the consumption of contaminated seafood may lead to poisoning with a wide range of symptoms, including a tingling sensation or numbness around the lips, nausea, vomiting, headaches, dizziness, diarrhea, visual disturbances, muscle paralysis, and myalgia [29,69]. Human poisoning caused by STXs has been repeatedly reported following the consumption of contaminated shellfish [161]. In severe poisoning, the muscular paralysis spreads, leading to death through respiratory paralysis [29,69]. Non-proteinogenic amino acid BMAA has been linked to a possible cause of the ALS/parkinsonism-dementia complex. BMAA has multiple effects on the translation process and bypasses the proof-reading ability of aminoacyl-tRNA-synthetase. BMAA may affect protein homeostasis and lead to mistranslation and misfolding of proteins in eukaryotic cells [162]. This neurotoxin has been found to alter gene expression and induce genotoxicity in nerve cells [74].

## 4. Cyanobacterial Toxin Analysis Techniques

Cyanotoxin analysis and monitoring are critical for ensuring water safety, particularly in recreational areas, as well as for assessing the potential risks associated with contaminated food products such as fish and shellfish, which can bioaccumulate toxins and act as an indirect exposure pathway for humans [30,163]. Various methods have been available to detect and identify cyanobacterial toxins in different matrices, including gas chromatography (GC) and high-performance liquid chromatography (HPLC) techniques, nuclear magnetic resonance (NMR), capillary electrophoresis (CE), bioassays, ELISA, protein phosphatase inhibition assay (PPIA), and molecular assays—polymerase chain reaction (PCR) and quantitative real-time PCR (qPCR). The available methodologies vary widely in terms of analytical sophistication and the scope of information obtained [30,162,163,164,165].

### 4.1. LC Methods

Liquid chromatography (LC) is an analytical technique that allows for the separation of compounds based on their interactions with the stationary and mobile phases, followed by detection using various types of detectors. Cyanotoxins are determined using reversed-phase liquid chromatography (RPLC) and hydrophilic interaction liquid chromatography (HILIC). In RPLC, the stationary phase is nonpolar (hydrophobic silica gels with grafted groups, such as C18), and the mobile phase is polar (mixtures of water and polar solvents such as acetonitrile and methanol). HILIC is an LC technique in which the stationary phase is polar (e.g., amine and silica gel), and the mobile phase is polar with a low water content. HILIC is preferred for polar compounds, such as BMAA and its congeners and STXs, which are often poorly retained in RPLC systems [57,166,167]. RPLC is commonly used for the analysis of MCs and NODs due to its robustness and suitability for moderately polar and nonpolar cyanotoxins [167]. LC-MS/MS is one of the most commonly employed LC methods and is considered a key analytical approach owing to its high sensitivity and selectivity, thereby enabling the precise identification of cyanotoxins at trace levels, including isomeric forms and the simultaneous determination of multiple cyanotoxin classes in complex environmental samples. There are several variations in LC-MS, including LC quadrupole time-of-flight (QTOF) mass spectrometry (LC-QTOF-MS) and LC-Orbitrap MS, each of which, in addition to the operating principle, differs in mass accuracy and resolution [168,169,170].

### 4.2. Biological Approaches to the Cyanotoxin Analysis

ELISA is an immunological technique based on a specific antigen–antibody reaction with toxins resulting in a change in the enzymatic activity of specific marks on the surface of antibodies. ELISA is significant in the context of cyanotoxin analysis due to its simplicity, cost-effectiveness, and rapid results. ELISA is widely used for routine monitoring of MCs and NODs in water [171]. Currently, commercial ELISA kits for detecting various toxins in water samples are available. One advantage of ELISA methods is the relative simplicity of use. However, ELISA does not provide information about the toxin profile, and a separate assay is required for each class [172,173].

PCR is a molecular biology technique used to amplify specific DNA sequences, allowing for detection and analysis of target genes or genetic markers even when they are present in very low concentrations in complex biological samples. PCR and qPCR are used to detect and quantify specific genes associated with cyanotoxin production, with their high sensitivity making these assays valuable tools for water quality monitoring as a method of early warning of potential cyanobacteria toxicity [174]. For example, Schembri et al. [175] used PCR techniques to study toxin production in *Raphidiopsis raciborskii* (formerly *Cylindrospermopsis raciborskii*).

PPIA is a rapid, sensitive colorimetric method with good reproducibility and low instrument requirements. PPIA is used to detect okadaic acid (OA) and dinophysistoxins [176] by measuring the extent to which a toxin sample reduces the activity of a purified protein phosphatase (PP1 or PP2A). It is also commonly employed to detect cyanotoxins MCs and NODs as these toxins are potent inhibitors of protein phosphatase [163,177]. An advantage of PPIA in cyanotoxin analysis is the assessment bioactivity, although toxin concentrations may be overestimated [178].

Biochemical-based and molecular methods are widely used to assess cyanobacterial toxicity. However, ELISA, PPIA, and PCR methods can be affected by sample interference, and unreliable results can occur. Furthermore, PPIA does not distinguish individual cyanotoxins. Therefore, it is important to use a technique such as LC-MS/MS for a comprehensive characterization of toxin profiles in samples from water bodies exposed to cyanoHABs [163,179].

### 4.3. Emerging Cyanotoxin Detection Systems

Developing user-friendly and compact detection systems (sensors) is essential for the on-site cyanotoxins monitoring. Cyanotoxin biosensors are devices that use biological molecules that specifically react with the target to detect and quantify cyanotoxins in different samples. Biosensors employ various technologies, including colorimetric, electrochemical, fluorescent, and plasmonic approaches [26,180]. A differential fluorescent sensor array implemented on a microfluidic chip allowing for identification and measurement of ATX-a, CYN, MC-LR and NOD in parallel has been developed [181]. The system is easy to use and enables simultaneous and rapid detection of multiple cyanotoxins. Li et al. [182] designed fluorescence-surface-enhanced Raman scattering (SERS) dual-modal biosensor for detection and quantification of MC-LR. Its advantages are accuracy and high selectivity to MC-LR detection. Electrochemical aptasensor modified with gold nanoparticles was designed to detect multiple cyanotoxins, MC-LR, CYN, ATX-a, STX and the algal toxin OA [183]. Bickman et al. [184] developed a portable biosensor system using a combination of fluorophore-conjugated monoclonal antibodies as detector molecules for the semiquantitative simultaneous screening of MC and CYN. Biosensors are primarily designed to analyze water samples, but methods are also being expanded to allow the analysis of other matrices [185]. A limitation of biosensors is their susceptibility to matrix interferences, particularly in complex samples [26,180]. Additionally, some biosensors require multiple washing steps [185]. More detailed information on cyanotoxin biosensors can be found in comprehensive reviews [180,185].

## 5. Simultaneous Cyanotoxin Detection

When considering analytical methods for cyanotoxin determination, most protocols and published methods, including EPA Methods 544 and 545 [186,187], focus on detection of specific toxin groups individually. HPLC-MS is extensively used for sensitive detection and accurate quantification of cyanotoxins in environmental and biological samples, including fruits and vegetables [188], as well as food supplements [165,189]. Well-developed methods exist for identifying MCs and NOD families, including those developed by Tran et al. [190] and Kaloudis et al. [191]. In addition, established methods are available for the detection of BMAA and its isomers [192,193], ATXs [194], and STXs [195,196]. The simultaneous detection of cyanotoxins from different classes is crucial for effective water quality and food monitoring to assess the toxic load.

### 5.1. MS-Based Techniques

Key challenges related to the comprehensive assessment of cyanobacterial toxins are their large structural diversity and different physicochemical properties, different sample preparation and unique chromatographic conditions [197]. In this regard, each class is typically analyzed separately. Due to their high polarity, STXs and BMAA are usually analyzed by HILIC techniques. But, in some cases, it is also possible to use reverse phase (RP) chromatographic methods implemented by adding a derivatization step to the sample preparation flow. For the analysis of BMAA, AEG and 2,4-DAB derivatization techniques, such as 6-aminoquinolyl-N-hydroxysuccinimidyl carbamate (AQC) [193], 9-fluorenylmethyl chloroformate (FMOC) [198], and ethylchloroformate (ECF) [199], are commonly used to increase molecular mass thereby enabling RP separation and improving ionization efficiency. The ECF derivatization procedure enables the conversion of BMAA into a nonpolar volatile compound, which is subsequently analyzed by GC [199]. Although AQC implies higher cost, it remains one of the most applied derivatization approaches due to its simplicity, reduced derivatization time (10–15 min), and good validation performance of methods employing this reagent [200,201,202,203]. This is further supported by the analysis of multiclass cyanotoxins in a recent study by Pravadali-Cekic et al. [204], who described an RP LC-MS/MS method for the simultaneous detection of BMAA, its isomers (2,4-DAB and AEG), and major microcystin congeners (MC-LR, MC-RR, MC-YR) including pre-column derivatization using AccQ-Tag Ultra Derivatization Kit (Waters, Milford, MA, USA) with linearity (R^2^ > 0.996) and accuracy (>90% recovery). Less polar cyanotoxins, which are cyclic peptides MCs and NOD, as well as alkaloids ATXs and CYNs, are analyzed by HPLC in the RP mode [168,205].

Simultaneous detection of different cyanotoxin classes, including multiple cyanotoxin analogues within the same class, is a challenging task that researchers are addressing using a variety of approaches. Table 2 details representative publications over the period 2015–2025 on the determination of multiclass cyanotoxins (two or more cyanotoxin classes) in environmental samples using LC-MS-based methods. A more detailed overview is provided in Supplementary Table S1. The combined use of HILIC and RP chromatography approaches also occurs and is implemented in many studies [167,206,207]. The used methods differ in instrumentation, sample preparation, and analysis configurations depending on the sample matrices and target cyanotoxins.

Modern RP LC-MS/MS methods can simultaneously analyze over 18 different cyanotoxins [208]. In the method reported by Jacinavicius et al. [208], MCs, NOD, ATX-a, hATX, CYN, deoxy-cylindrospermopsin (deoxy-CYN), GNT, and STXs were analyzed within acquisition times as short as 12 min using a 1290 series LC system coupled to a 6460—triple-quadrupole mass spectrometer (TQMS) from Agilent. The analysis monitored two transitions (quantifier and qualifier) using multiple reaction monitoring (MRM) in positive mode. However, the method did not allow for determining STX and dcSTX, as these compounds were not retained on the reverse-phase column employed. By contrast, Bergin et al. [57] developed an HILIC-MS/MS method for the simultaneous detection of STXs, ATXs, and MCs, allowing for separation of STX epimer pairs, including STX and dcSTX. The method enabled the acquisition of unique quantifier and qualifier MRM transitions required for reliable identification, compared with the RP LC-MS/MS method described above. The minimum LOD and LOQ values were between 0.00770 and 0.0485 μg L^−1^ and 0.0257 and 0.162 μg L^−1^ for ATXs, respectively; MCs showed intermediate sensitivity—LODs of 0.111–0.438 μg L^−1^ and LOQs of 0.369–1.46 μg L^−1^; for STXs, the detection limits were LODs of 0.0784–9.75 μg L^−1^ and LOQs of 0.261–32.5 μg L^−1^. All analytes demonstrated adequate linearity (R^2^ values ≥ 0.991) [57].

Due to its strong ability to retain highly polar analytes, the HILIC-MS/MS technique is a robust alternative to RP separations for multiclass analysis of cyanotoxins, such as ATXs, STXs, and CYN [57,215]. In contrast to RP columns, which often require ion-pairing strategies to retain very polar analytes, which may increase background noise and reduce ionization efficiency, HILIC provides improved retention and separation for hydrophilic compounds while maintaining compatibility with MS detection [206]. Recent HILIC-MS/MS methods have demonstrated low detection limits for multiclass cyanotoxins, including simultaneous determination of MCs (LODs of 0.0012–0.0021 μg L^−1^), NOD (LOD of 0.0021 μg L^−1^), CYN (LOD of 0.0012 μg L^−1^), ATX-a (LOD of 0.03 μg L^−1^), and BMAA with its congeners (LODs of 0.006–0.015 μg L^−1^) [166].

Configurations of LC-MS/MS systems coupled to a TQMS are widely used for the quantitative analysis of multiclass cyanotoxins, allowing for the obtention of results with sufficient sensitivity, low detection limits, and robust reproducibility [166,204,209,221]. Wang et al. [220] used an Agilent 1100 series LC system and an AB Sciex 4000 triple-quadrupole mass spectrometer to identify cyclic peptides, CYN and ATX-a, achieving LODs of 0.03–0.09 µg L^−1^. Even lower detection limits were reported by Zervou et al. [217], who analyzed water samples using a Finnigan Surveyor LC system (Thermo, USA) coupled to a TSQ Quantum Discovery Max triple-stage quadrupole mass spectrometer (Thermo, USA) operating in MRM mode. For all analytes, the detection limits ranged from 1 to 10 ng L^−1^, confirming high sensitivity of triple-quadrupole LC-MS/MS and its potential for further development in targeted quantification of multiclass cyanotoxins [217].

High-resolution mass spectrometry (HRMS) technique is also actively used in the study of multiclass cyanotoxins, ensuring high levels of selectivity, specificity, sensitivity, and reliable quantification [219]. Ultra-high performance liquid chromatography (UHPLC)-HRMS has emerged as a powerful tool, achieving method detection limits from pg L^−1^ to ng L^−1^ for multiclass cyanotoxins [142,143,215]. Filatova et al. [143] employed an Accela LC instrument (Thermo Fisher Scientific, San Jose, CA, USA) coupled to a Q-Exactive Orbitrap HRMS analyzer (Thermo Fisher Scientific, USA) with a heated electrospray ionization (HESI) source operating in the positive mode. For cyclic peptides such as MCs and NOD, HRMS-based methods frequently achieved LODs in the 4–150 pg L^−1^ range, whereas for CYN and ATXs, detection limits were 100 pg L^−1^ and 20 pg L^−1^, respectively [143]. The HRMS method is widely used for non-targeted analysis when certified standards are not available, allowing for the determination of a wide range of cyanobacterial metabolites simultaneously [223,228]. QTOF-MS systems can also be used for non-targeted analysis. For instance, Tatters et al. [223] utilized a UPLC I-Class coupled to a Xevo G2-XS QTOF-MS (Waters, Milford, MA, USA) in the RP mode for the non-targeted analysis of surface water samples. LC-HRMS systems using Orbitrap instruments are common [143,224]. Orbitrap-based MS systems provide high mass accuracy and resolving power, supporting reliable elemental composition and structural elucidation of closely related cyanotoxin congeners [143]. España Amórtegui et al. [168] used the HRMS/MS (Hybrid Q-Orbitrap) technique, achieving MS-resolved differentiation of structurally similar microcystin congeners such as MC-LR-[Dha7] and MC-LR-[Asp3]. Romera-García et al. [142] determined several MCs, CYN, ATX-a and hATX in water samples with method quantification limits (MDLs) of 8–45 ng L^−1^ and 25–129 ng L^−1^ for intracellular and extracellular cyanotoxins, respectively, employing Shimadzu Nexera UHPLC system (Den Bosch, the Netherlands) coupled to a Bruker Daltonics maXis 4G QTOF. Bogialli et al. [219] used a developed rapid LC-HRMS method to quantify target cyanotoxins and identify known variants using a suspect screening in water samples. The obtained results revealed, for the first time, the presence of uncommon cyanotoxins in Italian freshwater, proving that this protocol is well suited for strengthening surveillance programs for freshwaters affected by potentially toxic algal metabolites [219]. Compared with triple-quadrupole LC-MS/MS operated in the MRM mode, HRMS offers the advantage of full-scan acquisition, enabling retrospective data analysis and suspect screening [229,230]. However, for targeted quantification of well-characterized cyanotoxins, triple-quadrupole systems remain the most widely used [166,190,204,205,209,220,221].

Along with HPLC techniques using MS detection, capillary electrophoresis tandem mass spectrometry (CE-MS) methods show stable and good results in multiclass analysis of cyanotoxins [231,232]. Carmona-Molero et al. [231] have recently published a CE-MS method for determining a mixture of eight cyanotoxins belonging to three different classes: cyclic peptides (MC-LR, MC-RR, and NOD), alkaloids (CYN and ATX-a), and BMAA with its isomeric forms (2,4-DAB and AEG), achieving LODs within the range from 0.005 to 0.10 μg L^−1^ for MCs and NOD, 0.01 to 0.081 μg L^−1^ for alkaloids and 0.005 to 0.102 μg L^−1^ for BMAA and its congeners.

### 5.2. Sample Preparation

**Figure 2:**
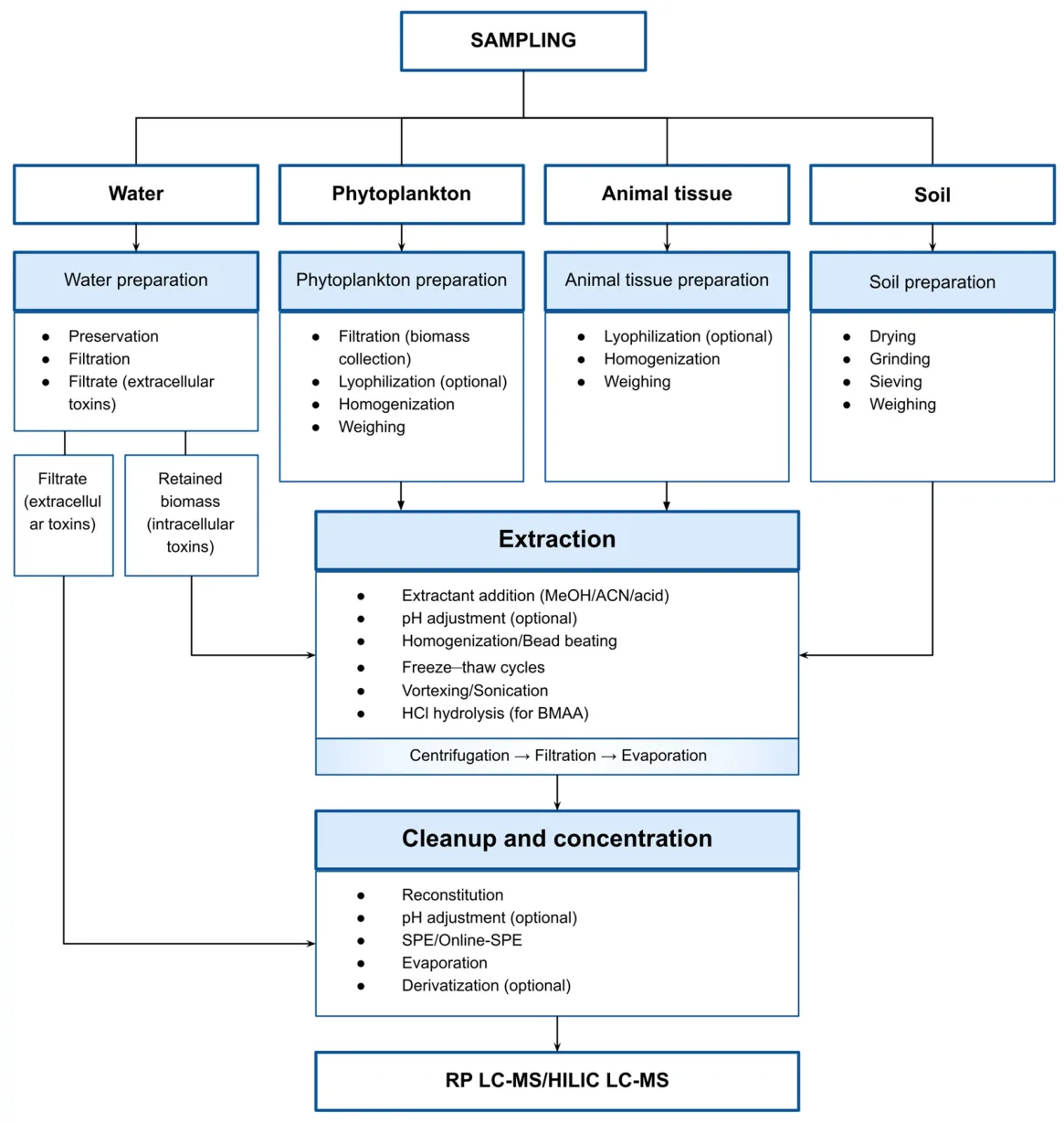
Sample preparation workflow for LC-MS cyanotoxin analysis.

Undoubtedly, sample preparation, including extraction, which is performed using an organic extractant such as methanol (MeOH), purification, and preconcentration steps, plays a key role in detecting cyanotoxins in environmental matrices using HPLC-MS [57,168,170,218]. A well-designed pre-analysis cleanup strategy can achieve desired results in terms of target analytes recovery and subsequent simultaneous detection. Sample preparation for detecting multiclass cyanotoxins varies depending on the type of matrix, the number and duration of steps and used equipment. The generalized sample preparation workflow for environmental samples is shown in Figure 2.

The most complex and labor-intensive sample preparation procedures are usually required for biological samples, such as animal tissues and phytoplankton, due to their complexity and multicomponent nature compared with water samples [179,190,207,210]. Typically, it involves intracellular and extracellular toxins extraction, which can be combined (total toxins extraction) or performed separately depending on the study objectives. For instance, Tran et al. [190], when working with aqueous samples, extracted extracellular and total cyanotoxins separately. For water samples, the sample preparation procedure is generally less complex, except for the step involving intracellular toxins extraction. The required volume of water is passed through a filter, typically 0.22–0.45 µm, after which the retained phytoplankton biomass on the filter surface undergoes further processing to extract intracellular toxins. This procedure is similar to that applied for the analysis of cyanobacterial biomass. Filter material impacts recovery. PTFE and PVDF are consistently identified as the most reliable materials for minimizing the adsorption of dissolved analytes [210,213]. Conversely, glass fiber (GF/F) filters may lead to losses, particularly for multiclass cyanotoxins, though they are useful for capturing biomass [224]. Intracellular toxin extraction is carried out in 2–3 steps and usually involves cell lysis, which includes various combinations of processes, such as freeze–thawing cycles, bead beating, vortexing, sonication, and centrifugation, in order to ensure release of toxins prior to HPLC analysis [57,168,217,218]. Freeze–thawing (typically −20 °C or −80 °C to room temperature) is highly effective and does not cause significant degradation of toxins [212,220]; for biomass, bead beating (using zirconia/silica beads) is faster and often provides superior disruption by finely dividing the sample, which enhances mass transfer into the solvent [142,212]; probe sonication is also used for rapid biomass disruption [208,210].

To achieve suitable extraction rates, various organic solvents are used and acidification of the extraction mixture is often carried out according to the physicochemical properties of the toxins, as well as the nature of the sample itself [207,210]. Recent HILIC-based multiclass LC-MS/MS methods demonstrated that extraction with 75–80% ACN acidified with 0.1% FA allows for efficient recovery of both moderately hydrophobic and highly polar toxin classes without additional partitioning steps [57,210]. In the research by Bergin et al. [57], an extraction mixture of 80:20 ACN:H_2_O acidified with 0.1% formic acid (FA) was used. Thomas et al. [210] extracted lyophilized cyanobacterial biomass with 75% ACN containing 0.1% FA. Such high organic solvent proportions simplify multiclass workflows, which is advantageous for routine applications. Christophoridis et al. [207] added n-BuOH during the final extraction step when using a MeOH:H_2_O (75:25, %) mixture. The addition of n-BuOH in the extraction workflow may improve recovery of less polar MC congeners, such as MC-LF and MC-LW. However, it increases the total solvent load and introduces an extra handling step, making the protocol more labor-intensive compared with single-solvent approaches. The use of acidic aqueous solutions (e.g., acetic acid) for polar toxins such as ATX-a [233] or CYN [227] is common but involves higher acid concentrations and may require additional neutralization prior to LC-MS analysis, as illustrated for ATX-a in Biré et al. [233]. In the study in [208], different approaches were used to extract different groups of cyanotoxins: MCs, NOD, and ATX-a were extracted with MeOH:H_2_O (1:1, *v*/*v*), and CYNs, STXs, and GNT were extracted with a 0.5 M acetic acid solution. This class-specific extraction strategy focuses on maximizing the extraction of each toxin group but requires several extraction procedures within a single workflow, increasing the sample preparation time and solvent consumption compared with unified multiclass protocols [208]. A similar approach was applied by Haddad et al. [206], who optimized the ACN:H_2_O ratio according to cyanotoxin polarity—25:75 ACN:H_2_O (*v*/*v*) for CYN, ATX-a and STX; 75:25 (*v*/*v*) ACN: 0.1% FA in H_2_O for MCs and NOD, achieving satisfactory recovery levels. In order to intensify lysis, commercial reagents are also used; in the study in [212], the lysing solution QuickLyseTM (Abraxis, Warminster, PA, USA) was used in one of the intracellular toxin extraction protocols enabling faster processing with acceptable results. However, this approach was less efficient than the traditional freeze–thaw method, but may be suitable in cases where rapid analysis is required [212].

It should be noted that, in addition to multiclass cyanotoxins, a well-designed sample preparation strategy can also enable the extraction of other cyanobacterial metabolites. Similar approaches can also be used for algal toxins, such as OA and DA [217]. A method has been reported that enables the determination of MCs, NOD, CYN, and ATXs, as well as DA [218]. In addition to cyanotoxins, You et al. [205] also determined anabaenopeptin A (AptA) and anabaenopeptin B (AptB), while Skafi et al. [214] additionally reported the determination of cyanopeptolin A. DA, OA, dinophysistoxin1, and dinophysistoxin2 can be determined together with a group of multiclass cyanotoxins using the method reported in Tatters et al. [223].

Effective cleanup and preconcentration are particularly important in multiclass cyanotoxin analysis. Solid-phase extraction (SPE) remains the predominant sample preparation technique for the purification and concentration of substances prior to HPLC analysis but alternative approaches to sample preparation that do not utilize SPE have also been reported [57,167,173,210]. SPE is typically used to enrich environmental concentrations of cyanotoxins, or to eliminate contaminants from complex samples such as animal and plant tissues [179,206,226]. To separate toxins with different physicochemical properties, various combinations of SPE can be used. The choice of sorbent in SPE cartridges (C18, based on polymeric cation exchange, graphitized carbon, etc.) for toxins extraction plays a crucial role in achieving optimal pre-concentration conditions and also influences primary parameters, such as selectivity, affinity, and capacity [142,234].

The most popular is the use of two SPE cartridges, the so-called dual SPE or tandem SPE. Aparicio-Muriana et al. [166], using a sample preparation strategy involving dual-SPE, developed a method for the determination of eight multiclass cyanotoxins—2 MCs, NOD, CYN, ATX-a, BMAA, 2,4-DAB, and AEG in aqueous samples. Strata-X Phenomenex and MCX SPE cartridges were used sequentially to separate first retaining low polar and moderately polar compounds, such as MCs, NOD, CYN, ATX-a, and then BMAA and its congeners, respectively [166]. The use of copolymer hydrophilic–lipophilic balanced Oasis HLB cartridges (Waters, USA) has been successful in a tandem SPE system in combination with HyperSep Hypercarb PGC (Thermo Scientific, UK) for water samples preparation to detect MCs, NOD, CYN, and ATX-a, as well as OA and DA [217]. Zervou et al. [216] extended the protocol to the analysis of not only cyanotoxins but also cyanopeptides (anabaenopeptins, microginins, aeruginosins and aeruginosamide) in Lake Vegoritis, Greece, employing dual system of cartridges Supel-Select HLB (200 mg, volume 6 mL, Supelco, St. Louis, MO, USA) and Supelclean ENVI-Carb (250 mg, volume 3 mL, Supelco, St. Louis, MO, USA) for the simultaneous extraction MCs, NOD, CYN, and ATX-a. Hammoud et al. [179] used a tandem SPE consisting of the same cartridge combination as in the study by Zervou et al. [216] to detect the same group of multiclass toxins, which confirms the effectiveness and reliability of the dual-SPE approach for multiclass cyanotoxin analysis in environmental matrices.

**Figure 3:**
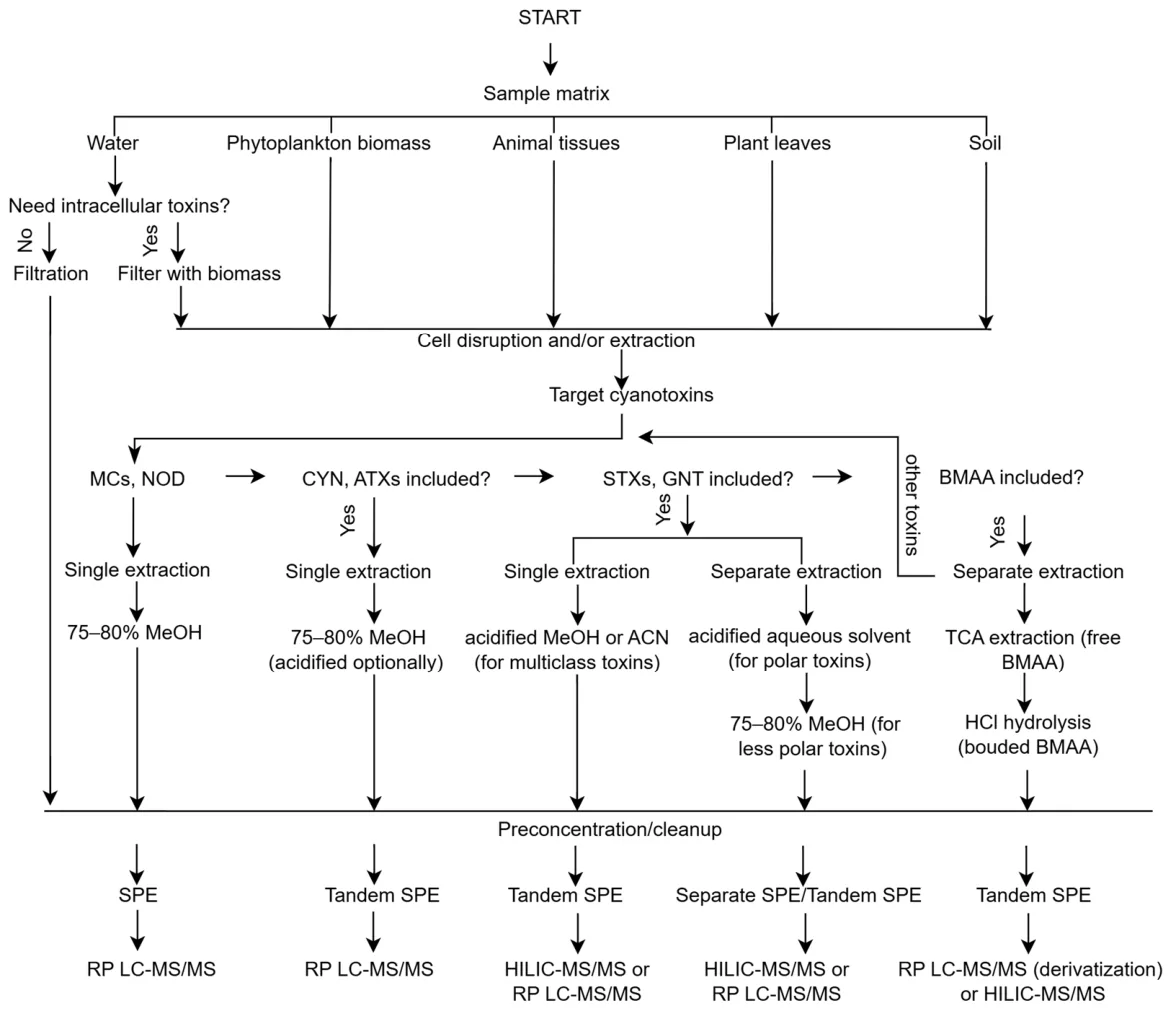
Decision tree for selecting analytical workflows for multiclass cyanotoxin determination according to sample matrix, target cyanotoxin classes, sample preparation strategy, and chromatographic separation.

In addition to conventional offline SPE and dual-SPE approaches, online SPE has also been used in cyanotoxin analysis. In online SPE, sample extraction and transfer to the analytical column are performed automatically within the chromatographic system, whereas offline SPE requires manual sample preparation steps prior to LC-MS analysis [235,236]. Strategies based on online-SPE sample preparation have an undeniable advantage—the reduction of the human factor influence and analyte loss due to the use of a larger number of replaceable containers where the solution and sample handling take place and the multi-step nature of the process itself [215,224]. The online-SPE method has been successfully implemented by Roy-Lachapelle et al. [224] for UHPLC-HRMS determination of 12 MCs, CYN, ATXs, AptA, and AptB, achieving an excellent extraction level for analytes with a total run time of 8 min. Online-SPE was also applied in [215] in combination with an HILIC-MS/MS method. For the determination of the multiclass toxins MCs, NOD, CYN and ATX-a, Fayad et al. [213] used online-SPE with a total run time of 7 min. Zhang et al. [225] successfully used this SPE variant for soil sample preparation. Compared with conventional offline-SPE, online-SPE significantly reduces the total analysis time while maintaining adequate sensitivity and satisfactory extraction efficiency for multiclass cyanotoxins. Direct coupling to the LC system minimizes sample handling steps, which contributes to improved reproducibility. In addition to reducing this step duration, the use of online-SPE helps reduce matrix effects and loss of target analytes during sample preparation. However, widespread use of this technique is limited by the complexity and high cost of the equipment, which is not always an affordable and optimal option for LC systems in regular laboratories designed for analyzing a wide range of chemical compounds. Based on the published studies reviewed in this article, Figure 3 presents a practical decision tree for selecting sample preparation strategies and LC-MS/MS workflows for multiclass cyanotoxin analysis based on the sample matrix and target cyanotoxin classes.

### 5.3. Current Challenges and Future Perspectives

Despite substantial progress in cyanotoxin analysis, several analytical and methodological challenges remain in the reliable detection and quantification of multiclass cyanotoxins [57,217,224]. Primary limitations are structural diversity and differences in physicochemical properties among classes of cyanotoxins, including variations in polarity, molecular weight, and functional groups, which complicate the development of universal LC-MS/MS methods capable of simultaneous multiclass analysis with sufficient sensitivity and selectivity [173,237]. This is clearly illustrated by methods that must accommodate cyclic peptides (MCs and NOD), alkaloids (CYN and ATX-a), and highly polar non-protein amino acids (BMAA, 2,4-DAB, and AEG) within a single LC-MS/MS workflow [166].

Along with the complexity of separating numerous analogues within several cyanotoxin families, another major limitation is the restricted availability of certified reference materials and isotopically labeled internal standards, particularly for emerging toxins such as newly identified AETX. Most ATX analogues also lack commercially available analytical standards [173]. Although more than 270 microcystin variants have been identified [58], only a small fraction of these variants is commercially available as certified reference materials [204,219]. This limits accurate quantification, method validation, and interlaboratory comparability.

Even when multiclass LC-MS-based methods are implemented, matrix effects and recovery variability remain significant limitations, especially when moving beyond water samples to more complex matrices and/or when aiming for low-level quantification [206]. In this context, sample preparation plays a key role. A vast range of physicochemical properties across toxin classes makes it difficult to develop a comprehensive extraction method, particularly for complex matrices such as phytoplankton biomass, animal tissues and soils, where toxins may strongly bind to organic matter or clay minerals [166,168,225]. Dual-cartridge or tandem cleanup strategies have been successfully applied to improve multiclass coverage and sensitivity in water monitoring [143]. Advances in automation and the coupling of extraction techniques to LC-MS/MS, including online-SPE-LC-MS/MS, can reduce the number of steps in sample preparation and manual handling and enable rapid analysis [213]. Nevertheless, the issue of reducing the use of toxic solvents and developing simplified workflows remains relevant, for example, through the application of solid-phase-enhanced sample preparation (SP3), which is widely used in proteomics [238]. In this light, QuEChERS-based strategies (it stands for “quick, easy, cheap, effective, rugged and safe”) which utilize dispersive solid-phase extraction (dSPE) can improve analytical performance in complex environmental matrices [237].

Future research should focus on the development of standardized multiclass analytical protocols integrating optimized sample preparation and advanced LC-MS/MS and HRMS techniques [239]. In the absence of reference standards, HRMS enables the detection of emerging toxins and unknown cyanotoxin analogues based on accurate mass and fragmentation patterns [237]. The combination of target, suspect, and non-target screening approaches is expected to improve the detection of known and emerging cyanotoxins. Recent advances in HRMS, including QTOF and Orbitrap systems, enable suspect screening and non-target analysis, allowing for the detection of unknown cyanotoxins and transformation products [228,239]. Roy-Lachapelle et al. [228] have shown that integrating targeted, suspect, and non-target analyses significantly expands the identification of uncommon and novel cyanopeptide congeners. Data-independent acquisition HRMS strategies can further support structural characterization and the discovery/annotation of uncommon or previously unreported cyanopeptides, strengthening future multiclass monitoring [240]. These approaches will provide new opportunities for comprehensive multiclass cyanotoxin profiling but require further development of spectral libraries, data processing workflows, and identification criteria [237,239].

## 6. Conclusions

Cyanobacteria produce a wide range of toxic metabolites, often simultaneously. The accumulation of cyanotoxins in aquatic environments and biota poses significant risks to human and animal health. This review highlights that the co-occurrence of multiple cyanotoxin classes is common and requires reliable analytical approaches for their timely detection. Currently, many established and validated methods have demonstrated LODs and LOQs well below regulated toxin limits. For routine targeted analysis, triple-quadrupole LC-MS/MS remains the preferred approach. LC-HRMS has considerably improved multiclass cyanotoxin analysis, enabling the separation of related cyanotoxin structural variants and non-target screening. The greatest analytical challenge remains the simultaneous determination of highly polar cyanotoxins such as STXs and BMAA together with less polar cyanotoxins such as MCs and NOD because of their different physicochemical properties, thereby requiring different sample preparation strategies and chromatographic conditions. Matrix nature poses additional challenges for multiclass analysis, particularly in tissues and cyanobacterial biomass. Although online-SPE has shown good results in terms of reducing analyte loss and matrix effects, it is not commonly applied due to its technical complexity. Nevertheless, a wide range of analytical strategies are being proposed for the simultaneous analysis of multiple cyanotoxin groups, including those designed for the extraction of other toxins, such as DA, expanding the scope of application of the LC-MS/MS approach. To facilitate multiclass cyanotoxin analysis method selection, this review proposes a practical decision tree that summarizes analytical workflows discussed in the reviewed literature. However, despite significant progress in cyanotoxin monitoring, there is still no unified approach for the simultaneous determination of multiple cyanotoxin classes. Therefore, the development of reliable, sensitive, and cost-effective analytical approaches capable of covering a broad spectrum of cyanotoxins remains essential for effective environmental monitoring and risk assessment.

## Figures and Tables

**Table 1:** Cyanobacterial genera associated with different classes of cyanotoxins. The “+” symbol indicates the potential to produce the respective class of cyanotoxins.

Genera	MCs	NODs	CYNs	ATXs	STXs	GNT	BMAA	References
*Microcystis*	+			+			+	[1,74]
*Dolichospermum*	+		+	+	+	+	+	[1,74]
*Nostoc*	+	+					+	[1,74,76]
*Nodularia*		+					+	[1,74]
*Kamptonema*	+		+	+				[1,84,85,86]
*Phormidium*	+			+	+		+	[1,74,79]
*Planktothrix*	+			+	+		+	[1,74]
*Microcoleus*	+			+			+	[1,74,78]
*Fischerella*	+						+	[1,74]
*Aphanizomenon*			+	+	+		+	[1,74]
*Raphidiopsis*	+		+	+	+		+	[1,74,87]
*Microseira*			+		+			[1]
*Cylindrospermum*				+	+			[1,79]
*Scytonema*					+		+	[1,74]
*Anabaenopsis*	+						+	[1,74]
*Synechococcus*	+						+	[1,74]
*Calothrix*	+						+	[1,74]
*Cuspidothrix*				+	+			[88,89,90]
*Tychonema*	+			+				[91,92,93,94]
*Synechocystis*	+						+	[74,86,95]
*Anagnostidinema*	+			+	+			[79,96,97]
*Pseudanabaena*	+			+	+		+	[74,86,98,99]
*Toxifilum*	+				+			[98]
*Oscillatoria*	+		+	+			+	[74,100,101,102]
*Limnospira*	+			+				[103,104,105]
*Leptolyngbya*	+				+			[106,107]
*Trichodesmium*	+			+	+		+	[74,108,109]

**Table 2:** LC-MS-based methods for the simultaneous determination of multiclass cyanotoxins.

No	Sample Type	Sample Preparation				Detection Techniques	Target Analytes	LOD	LOQ	Reference
		Freeze–Thaw	Extraction	SPE	Specific Features					
1.	Cyanobacterial biomass	Yes	80% ACN with 0.1% FA	-	-	HILIC-MS/MS	3 MCs, 11 STXs, ATX-a, hATX	0.0077–9.75 μg L^−1^	0.0257–32.5 μg L^−1^	[57]
2.	Cyanobacterial biomass	-	50% MeOH (for MCs, NOD, ATX); 5 M acetic acid (for CYNs, STXs, GNT)	-	-	LC-MS/MS	7 MCs, NOD, ATX-a, hATX, CYN, deoxy-CYN, GNT, 9 STXs	NA	NA	[208]
3.	Cyanobacterial biomass	Yes	50% MeOH with 0.1% FA	-	-	LC-MS/MS	17 MCs, 3 CYNs, 17 ATXs, NOD-R, AETX	0.11–2.8 ng g^−1^	NA	[173]
		-	MeOH (% not specified)	-	SPATT (HP-20 resin) used					
4.	Cyanobacterial biomass	-	75% MeOH	-	-	HPLC-ESI-MS/MS	7 MCs, NOD, STX, ATX-a	0.0001–0.001 μg mL^−1^	0.0005–0.005 μg mL^−1^	[98]
5.	Scum	Yes	10% TCA (for BMAA)	-	6M HCl hydrolysis; derivatization	LC-MS/MS	3 MCs, BMAA, 2,4-DAB, AEG	0.13–0.46 ng mL^−1^	0.85–1.38 ng mL^−1^	[204]
		-	70% MeOH (for MCs)	-	-					
6.	Algal biomass	Yes	-	-	-	UPLC-MS/MS	8 MCs, NOD, CYN, ATX-a	NA	NA	[209]
7.	Shellfish tissue	-	MeOH (% not specified)	Tandem SPE: C18 Bakerbond (500 mg, 6 mL, Dicsa, Andalucía, Spain) and graphitized carbon BOND ELUT^®^ (500 mg, 6 mL, Agilent, Amstelveen, the Netherlands)	pH 11 adjusting before SPE	UPLC-MS/MS	3 MCs, CYN	0.01–0.39 ng g^−1^ fw	0.23–0.40 ng g^−1^ fw	[65]
8.	Shellfish tissue	-	100% MeOH	-	-	LC-MS/MS (QQQ)	13 MCs, NOD, ATX, hATX, CYN	2.1–4.04 μg kg^−1^	6.29–12.11 μg kg^−1^	[168]
	Cyanobacterial biomass	Yes				LC-HRMS/MS (Hybrid Q-Orbitrap)	MC-LR-[Dha7], MC-LR-[Asp3]	NA	NA	
9.	Cyanobacterial biomass, shellfish tissue	-	75% ACN with 0.1% FA	-	-	HILIC-MS	17 MCs, NOD-R, ATX-a, hATX, CYN, deoxyCYN, 14 STXs, GNT, LWTX1	0.01–0.99 μg g^−1^	NA	[210]
10.	Water, scum, foam, cyanobacterial biomass	-	-	-	Direct injection	LC-MS	11 MCs, NOD-R, CYN, ATX-a	* 0.05–0.28 ng mL^−1^	NA	[211]
		Not detailed				UPLC-MS/MS		<0.5 ppb	NA	
						LC-MS/SIM		0.05 ng g^−1^	NA	
		Yes	Acidified MeOH	-	-	LC-MS/MS		NA	NA	
11.	Water, cyanobacterial biomass	-	75% MeOH	-	Pellet re-extraction (2 times, last–with n-BuOH)	LC-MS/MS (for MCs, NOD, CYN, ATX-a)	12 MCs, NOD, CYN, ATX-a, STX, neoSTX	0.1–1.0 ng mg^−1^ dw	0.4–2.9 ng mg^−1^ dw	[207]
		-	-	Tandem SPE (not detailed)	-			0.8–6.5 ng L^−1^	NA	
		-	-	-	Direct injection	HILIC-MS (for STXs)		0.1–3 µg L^−1^	NA	
12.	Water, scum	Yes	Different extraction protocols tested	-	Lysing solution QuickLyseTM (Abraxis, Warminster, PA, USA) used	UPLC-MS/MS	17 MCs, NOD, CYN, deoxy-CYN, ATX-a, hATX	NA	0.1–0.5 µg L^−1^	[212]
13.	Water, scum	Yes	-	-	pH 2 adjusting	Online-SPE-LC-HESI-MS/MS	6 MCs, NOD, CYN, ATX-a	* 0.01–0.02 µg L^−1^	** 0.03–0.07 µg L^−1^	[213]
14.	Water	-	-	Supelclean ENVI-carb (500 mg, 6 mL, Supelco, Bellefonte, PA, USA) (for CYN, ATX-a, STX) and Oasis HLB (200 mg, 6 mL, Waters, Milford, MA, USA) (for MCs and NOD)	-	LC-MS/MS	5 MCs, NOD, ATX-a, CYN, STX	0.004–0.07 ng mL^−1^	0.01–0.23 ng mL^−1^	[206]
	Fish tissue		25% ACN (for CYN, ATX-a, STX); 75% ACN with 0.1% FA (for MCs, NOD)					0.02–0.08 ng mL^−1^	0.07–0.28 ng mL^−1^	
15.	Water	-	Not detailed	-	-	Online-SPE–UHPLC-MS/MS	12 MCs, ATX-a, hATX, CYN, cyanopeptolin A, anabaenopeptins A and B (AptA, AptB)	0.1–10 μg kg^−1^	0.3–33 μg kg^−1^	[214]
	Fish tissue		MeOH (% not specified)							
16.	Water	-		Tandem SPE: Supel-Select HLB (200 mg, 6 mL, Supelco) and Supelclean ENVICarb (250 mg, 3 mL, Supelco)	pH 11 adjusting before SPE	LC-MS/MS	12 MCs, NOD, CYN, ATX-a	0.001–0.007 μg L^−1^	0.003–0.02 μg L^−1^	[179]
			75% MeOH (for filter with biomass)	-	-					
	Fish tissue	-	80% MeOH, hexane	Supel-Select HLB (200 mg, 6 mL, Supelco)	-					
17.	Water	Yes	-	-	Filtrate analysis	RP-UPLC-MS/MS	17 MCs, NOD, ATX-a, hATX, CYN	NA	0.1 μg L^−1^	[167]
						UPLC-HILIC-MS/MS	STX, dcSTX, dcneoSTX	NA		
18.	Water	-	-	ENVI-Carb (PGC, 250 mg, 3 mL, Supelco, Bellefonte, PA, USA)	For STXs only	Online-SPE-HILIC-MS/MS	ATX-a, CYN, 13 STXs	0.7–4.1 ng L^−1^	1.8–8.1 ng L^−1^	[215]
					-	HILIC-MS/MS		1.1–54.5 ng L^−1^	2.5–130.4 ng L^−1^	
19.	Water	Yes	-	-	Direct injection (for CYN, ATX-a, hATX)	LC-MS/MS	12 MCs, CYN, ATX-a, hATX, NOD, anabaenopeptin A (AptA) and anabaenopeptin B (AptB)	* 0.02–33 ng L^−1^	** 0.06–100 ng L^−1^	[205]
				Oasis HLB (200 mg, 6 mL, Waters, USA) (for MCs, NOD, AptA, AptB)	-					
20.	Water	-	-	Tandem SPE: Strata-X cartridges (Phenomenex, Torrance, CA, USA) and MCX different sorbent mass (60 mg and 150 mg, Waters, Milford, MA, USA)	-	HILIC-MS/MS	MC-LR, MC-RR, NOD, CYN, ATX-a, BMAA, 2,4-DAB, AEG	0.0012–0.03 μg L^−1^	0.004–0.1 μg L^−1^	[166]
21.	Water	-	-		Direct injection	UHPLC-HRMS	6MCs, CYN, ATX-a, hATX	* 3–45 ng L^−1^	** 8–45 ng L^−1^	[142]
			75% MeOH (for filter with biomass)		-					
22.	Water	-	-	Tandem SPE: Supel-Select HLB (200 mg, 6 mL, Supelco, St. Louis, MO, USA) and Supelclean ENVI-Carb (250 mg, 3 mL, Supelco, St. Louis, MO, USA)	pH 11 adjusting before SPE	LC-MS/MS	12 MCs, NOD, CYN, ATX-a, and 10 cyanopeptides (3 APs, 4 MGs, 2 AERs and aeruginosamide)	0.001–0.007 μg L^−1^	0.003–0.02 μg L^−1^	[216]
			75% MeOH (for filter with biomass)	-	-					
23.	Water	-	-	Tandem SPE: Oasis HLB (500 mg, 6 mL, Waters, Milford, MA, USA) and Supelclean ENVI-Carb (500 mg, 6 mL, Supelco, St. Louis, MO, USA)	-	UHPLC-HRMS	7 MCs, CYN, ATX-a, NOD	4–150 pg L^−1^	12–450 pg L^−1^	[143]
24.	Water	-	-	Tandem SPE: Oasis HLB (200 mg, 6 mL, 25–35 µm, Waters, USA) and HyperSep Hypercarb PGC (200 mg, 3 mL, 30–40 µm, Thermo Scientific, UK)	pH 11 adjusting before SPE	LC-MS/MS	12 MCs, NOD, CYN, ATX-a	1–10 ng L^−1^	NA	[217]
25.	Water	yes	-	-	Hemoflow cartridge (60 Da exclusion size) used	UPLC-MS/MS	3 MCs, NOD, CYN, ATX-a, hATX, dihydro derivatives of ATX-a (H_2_-homoATX-a, H_2_-ATX-a)	0.0038–0.0078 μg L^−1^	0.01–0.02 μg L^−1^	[218]
26.	Water	Not detailed				LC-QTOF HRMS	9 MCs, NOD, CYN, ATX-a and standardless cyanotoxins from database	NA	NA	[219]
27.	Water	-	-	-	Supernatant analysis	LC-MS/MS	8 MCs, NOD, CYN, ATX-a	0.03–0.09 µg L^−1^	0.1–30 µg L^−1^	[220]
		Yes								
28.	Water	-	-	-	Filtrate analysis	LC-MS/MS	6 MCs, NOD, CYN, ATX-a	0.04–0.8 mg L^−1^	0.1–2.3 mg L^−1^	[221]
			100% MeOH (for filter with biomass)		Pellet re-extraction 75% MeOH					
29.	Water	-	-	-	Direct injection	LC-MS/MS	CYN, ATX-a, hATX	* 15–70 ng L^−1^	** 50–240 ng L^−1^	[190]
		Yes			-					
		-	-	Chromabond^®^ HR-X (500 mg, 6 mL, Macherey-Nagel), Oasis HLB (200 mg, 6 mL, Waters), Supelclean Coconut Charcoal (2000 mg, 6 mL, Supelco)	LOD/LOQ for CYN not determined (low recovery)			* 0.6–1.3 ng L^−1^	** 2–4 ng L^−1^	
30.	Water	Yes	ACN:H_2_O:FA (80:19.9:0.1, *v*/*v*) (for filter with biomass)	-	Reconstitution with 50% MeOH (for MCs)	LC-MS/MS	8 MCs, CYN, deoxy-CYN, ATX-a, 12 STXs	NA	0.04–0.5 µg L^−1^	[83]
					Reconstitution with 75% ACN (for STXs)			NA	0.01–0.02 µg L^−1^	
					Reconstitution with 0.1% FA (for CYNs, ATX-a)			NA	0.1–2 µg L^−1^	
31.	Water	Yes	75% MeOH	-	-	LC-MS/MS	5 MCs, ATX-a	NA	NA	[222]
32.	Water	Yes	-	-	Filtrate analysis	UPLC-MS/MS	11 MCs, ATX-a, CYN, deoxy-CYN, AptA, AptB, euglenophycin	0.5 µg L^−1^	NA	[223]
		-	Not detailed	-	SPATT: DIAION HP20	LC-MS/SIM	5 MCs, NOD-R, ATX-a, hATX, CYN	<0.5 ng g ^−1^	NA	
33	Water	Yes	-	-	-	Online-SPE-UHPLC-HRMS	12 MCs, CYN ATXs, AptA, AptB	* 8–53 ng L^−1^	** 27–176 ng L^−1^	[224]
34.	Soil	-	MeOH with 200 mM ammonium acetate	-	-	Online-SPE-UHPLC-MS/MS	10 MCs, NOD-R, ATX-a, hATX, CYN, AptA, AptB, cyanopeptolin A	0.001–0.3 ng g^−1^	0.003–0.9 ng g−1	[225]
35.	Plant leaves	-	80% MeOH	Tandem SPE: C18 Bakerbond^®^ (500 mg, 6 mL, Dicsa, Andalucía, Spain) and BOND ELUT^®^ Carbon cartridge (Agilent, Amstelveen, the Netherlands)	-	UPLC-MS/MS	3 MCs, CYN	0.06–0.42 ng g^−1^ fw	0.16–0.91 ng g^−1^ fw	[226]
36.	Plant leaves	-	80% MeOH (for MCs, CYN)	Tandem SPE: Bakerbond^®^ C18 (Dicsa, Andalucía, Spain) and Bond Elut Carbon PGC (Agilent, Santa Clara, CA, USA)	pH 11 adjusting before SPE	UPLC-MS/MS	3 MCs, CYN	NA	NA	[227]
			10% acetic acid (for samples spiked with CYN)	Bond Elut Carbon PGC (Agilent, Santa Clara, CA, USA)						

Abbreviations: MCs—microcystins, MC-—microcystin-, NOD—nodularin, NOD-R—nodularin-R, GNT—guanitoxin, AptA—anabaenopeptin A, AptB—anabaenopeptin B, LWTX1—lyngbyatoxin 1, ATXs—anatoxins, ATX-a—anatoxin-a, hATX—homoanatoxin, CYNs—cylindrospermopsins, CYN—cylindrospermopsin, deoxy-CYN—deoxy-cylindrospermopsin, AETX—aetokthonotoxin, STXs—saxitoxin analogues, STX—saxitoxin, neoSTX—neosaxitoxin, dcneoSTX—decarbamoylneosaxitoxin, dcSTX—decarbamoylsaxitoxin, BMAA—β-methylamino-L-alanine, 2,4-DAB—2,4-diaminobutyric acid, AEG—aminoethyl glycine, fw—fresh weight, dw—dry weight, HILIC—hydrophilic interaction liquid chromatography, HPLC-MS—high-performance liquid chromatography mass spectrometry, LC-MS—liquid chromatography mass spectrometry, LC-MS/MS—liquid chromatography-tandem mass spectrometry, UHPLC-MS/MS—ultra-high performance liquid chromatography-tandem mass spectrometry, RP-UPLC-MS/MS—reverse-phase ultra-performance liquid chromatography-tandem mass spectrometry, HRMS—high-resolution mass spectrometry, SPE—solid-phase extraction, ACN—acetonitrile, MeOH—methanol, FA—formic acid, SPATT—solid-phase adsorption toxin tracking (passive sampler), PGC—porous graphitic carbon. * Method detection limit (MDL). ** Method quantification limit (MQL).

## Data Availability

No new data were created or analyzed in this study.

## Supplementary Materials

The following supporting information can be downloaded at: https://www.mdpi.com/article/10.3390/toxins18090395/s1, Table S1: LC-MS-based methods for the simultaneous determination of multiclass cyanotoxins.

## Abbreviations

The following abbreviations are used in this manuscript:

CyanoHABs	Cyanobacterial harmful algal blooms
MCs	Microcystins
MC-LR	Microcystin-LR
MC-RR	Microcystin-RR
MC-YR	Microcystin-YR
ADDA	(2*S*,3*S*,4*E*,6*E*,8*S*,9*S*)-3-Amino-9-methoxy-2,6,8-trimethyl-10-phenyldeca-4,6-dienoic acid
NOD	Nodularin
NODs	Nodularins
NOD-R	Nodularin-R
CYN	Cylindrospermopsin
CYNs	Cylindrospermopsins
ATX-a	Anatoxin-a
ATXs	Anatoxins
hATX	Homoanatoxin
AETX	Aetokthonotoxin
STXs	Saxitoxins
PSP	Paralytic shellfish poisoning
neoSTX	Neosaxitoxin
BMAA	ß-Methylamino-L-alanine
2,4-DAB	2,4-Diaminobutyric acid
AEG	Aminoethyl glycine
ELISA	Enzyme-linked immunosorbent assay
PCR	Polymerase chain reaction
qPCR	Quantitative polymerase chain reaction
PPIA	Protein phosphatase inhibition assay
RP	Reverse-phase
RPLC	Reversed-phase liquid chromatography
HILIC	Hydrophilic interaction liquid chromatography
HPLC-MS	High-performance liquid chromatography mass spectrometry
LC-MS	Liquid chromatography mass spectrometry
LC-MS/MS	Liquid chromatography-tandem mass spectrometry
RP-UPLC-MS/MS	Reverse-phase ultra-performance liquid chromatography-tandem mass spectrometry
HRMS	High-resolution mass spectrometry
TQMS	Triple-quadrupole mass spectrometer
HESI	Heated electrospray ionization
ACN	Acetonitrile
MeOH	Methanol
FA	Formic acid
n-BuOH	n-Butanol
dw	Dry weight
fw	Fresh weight
ww	Wet weight
