# Supplementary material for: Multiclass Cyanotoxins in Aquatic Environments: Occurrence, Impacts and Detection Techniques
Source: Toxins (Basel). 2026 Sep 14;18(9):395. doi: 10.3390/toxins18090395 (PMC13611334; doi:10.3390/toxins18090395)
Supplement: Supplementary file 1 [file toxins-18-00395-s001.zip › toxins-4404977-supplementary.pdf]

Review

Supplementary Materials: Multiclass cyanotoxins in aquatic environments: occurrence, impacts and detection techniques

Galina Nugumanova

Table S1. LC-MS-based methods for the simultaneous determination of multiclass cyanotoxins [a].

№	Sample Type	Sample Preparation	Detection Techniques	Chromatographic Separation Conditions	Target Analytes	LODs	LOQs	Reference
1.	Cyanobacterial biomass	Lysis (freeze–thaw)–lyophilization–weighing 10–50 mg–extraction 80:20 ACN:H_2O with 0.1% FA–sonication 20 min–centrifugation–filtrate collection	HILIC-MS/MS	Column: Waters BEH Amide column (2.1 × 100 mm, 1.7 μm)<br>Temperature: 40◦C<br>Flow rate: 0.5 mL min−1<br>Mobile phases: A—2 mM ammonium formate in H_2O at a pH of 3.5, B—0.25% FA in ACN<br>Injection volume: 1 μL<br>Total run time: 25 min	3 MCs, 11 STXs, ATX-a, hATX	0.0077 – 9.75 μg L−1	0.0257 – 32.5 μg L−1	[57]
2.	Cyanobacterial biomass	Lyophilization–weighing 50 ± 0.05 mg–2 steps extraction: 1) for MCs, NOD, ATX–MeOH:H_2O (1:1, v/v, 2) for CYNs, STXs, GNT–0.5 M acetic acid–probe sonication at 30% amplitude for 3 min–bead beating 1 min–centrifugation–filtration, PVDF filter 0.22 μm	LC-MS/MS	Column: Synergy Hydro-RP 80 A column (150 × 2.0 mm, 4 μm, Phenomenex, USA)<br>Temperature: 30 °C<br>Flow rate: 0.4 mL/min<br>Mobile phases: A—H_2O with 0.1% (v/v) FA and 2 mM ammonium formate, B—ACN/H_2O (8:2, v/v) with 0.1% (v/v) FA<br>Injection volume: 10 μL<br>Total run time: 12 min	7 MCs, NOD, ATX-a, hATX, CYN, deoxy-CYN, GNT, 9 STXs	NA	NA	[208]
3.	Cyanobacterial biomass	Lysis (freeze–thaw) / lyophilization–weighing–extraction MeOH:H_2O (1:1, v/v) containing 0.1% (v/v) FA–centrifugation–filtration, PVDF filter 0.45 μm;<br>SPATT: 2 g of wet activated HP-20 resin from sampler was extracted with MeOH using an empty SPE cartridge with a frit–drying–dilution with MeOH:H_2O (1:1, v/v) containing 0.1% (v/v) FA–centrifugation–filtration, PVDF filter 0.45 μm	LC-MS/MS	Column: Acquity HSS T3 column (150 × 2.1 mm, 1.8 μm) fitted with a VanGuard Acquity HSS T3 pre-column (5 × 2.1 mm, Waters, USA)<br>Temperature: 40 °C<br>Flow rate: 0.42 – 0.55 mL/min<br>Mobile phases: A—0.1% (v/v) FA in H_2O, B—0.1% (v/v) FA in ACN<br>Injection volume: 1 μL<br>Total run time: 30 min	17 MCs, 3 CYNs, 17 ATXs, NOD-R, AETX	0.11 – 2.8 ng g −1	NA	[173]

4.	Cyanobacterial biomass	Lyophilization–extraction 2.5 mL of 75 % MeOH at 45 °C (3 times)–evaporation–centrifugation–reconstitution with 900 µL MeOH–filtration, cellulose-acetate filter 0.2 µm	HPLC-ESI-MS/MS	Column: Agilent Eclipse Plus C18 (2.1 × 100 mm, 1.8 µm)<br>Temperature: 40 °C<br>Flow rate: 0.5 mL/min<br>Mobile phases: A—0.1% FA in H_2O, B—0.1% FA in ACN<br>Injection volume: 20 µL<br>Total run time: 16 min	7 MCs, NOD, STX, ATX-a	0.0001 – 0.001 µg mL –1	0.0005 – 0.005 µg mL –1	[98]
5.	Scum	BMAA: lyophilization–weighing 15 mg–lysis (10% TCA)–probe sonication for 1 min–overnight precipitation at 4 °C–centrifugation–supernatant collection–repeat lysis 3 times, 3rd time 10% TCA/acetone used–100% acetone flushing of pellet in a shell vial–centrifugation–supernatant collection–combined supernatant concentration (free fraction)–6M HCl hydrolysis of pellet (bound fraction)–filtration, filter 0.2 µm<br>MCs: lyophilization–weighing ~5 mg–extraction 70 % MeOH–probe sonication for 1 min–centrifugation–decantation–filtration, filter 0.2 µm–pellet re-extraction (2 times)–combined supernatant centrifugation–AccQ-Tag derivatization of combined BMAA and MCs exstracts	LC-MS/MS	Column: Zorbax RRHD Eclipse Plus C18 column (2.1 × 100 mm, 1.8 µm, Agilent)<br>Temperature: 18 °C<br>Flow rate: 0.65 mL min –1<br>Mobile phases: A—0.1% v/v FA in H_2O, increasing to 10% B—0.1% v/v FA in ACN<br>Injection volume: 5 µL<br>Total run time: 14 min	3 MCs, BMAA, 2,4-DAB, AEG	0.13 – 0.46 ng mL–1	0.85 – 1.38 ng mL–1	[204]
6.	Algal biomass	Frozen biomass samples–lysis (freeze–thaw cycles, 2 times)–filtration, GHP filter 0.45 mm	UPLC-MS/MS	Column: Acquity UPLC HSS T3 column (100 mm x 2.1 mm, 1.8 µm, 130 Å, Waters)<br>Temperature: 35 °C<br>Flow rate: 0.45 mL/min.<br>Mobile phases: A—0.1% FA in H_2O, B—0.1% FA in ACN<br>Injection volume: 5 µL<br>Total run time: 12 min	8 MCs, NOD, CYN, ATX-a	NA	NA	[209]
7.	Shellfish tissue	Tissue homogenization–weighing 1.0 g–lyophilization–extraction MeOH–sonication–stirring–centrifugation–supernatant collection–supernatant concentration–MeOH dilution–pH 11 adjusting–tandem SPE, cartridges C18 Bakerbond (500 mg, 6 mL, Dicsa, Spain) and graphitized carbon cartridges BOND ELUT® (500 mg, 6 mL, Agilent, the Netherlands)–evaporation–reconstitution with 1 mL 20% MeOH	UPLC-MS/MS	Column: XSelect HSS T3 column (100 × 2.1 mm, 2.5 µm)<br>Flow rate: 0.45 mL min−1<br>Mobile phases: A—0.1% FA in H_2O, B—0.1% FA in ACN<br>Injection volume: 5 µL<br>Total run time: 10 min	3 MCs, CYN	0.01 – 0.39 ng g −1 fw	0.23 – 0.40 ng g −1 fw	[65]
8.	Shellfish tissue	Tissue: tissue homogenization–weighing 2.0 g–extraction 100 % MeOH–vortexing 2 min–centrifugation–filtration, filter 0.22 µm–dilution 1:4, with H_2O, v/v; phytoplankton: filter with biomass–lysis (freeze–thaw)–extraction 100 % MeOH–vortexing 2 min–shaking 20 min–filtration, filter 0.22 µm–dilution 1:4, with H_2O, v/v	LC-MS/MS (QQQ)	Column: ACQUITY BEH C18 UPLC column (2.1 x100 mm, 1.7 µm, Waters, UK) fitted with a VanGuard ACQUITY BEH C18 UHPLC pre-column (2.1 mm x 5 mm, 1.7 µm, Waters, UK)<br>Temperature: 60 °C	13 MCs, NOD, ATX, hATX, CYN	2.1 – 4.04 µg kg –1	6.29 – 12.11 µg kg –1	[168]

				Flow rate: 0.4 mL/min<br>Mobile phases: A—0.025% (v/v) FA in H_2O, B—0.025% (v/v) FA in ACN<br>Injection volume: 5 μL<br>Total run time: 11 min				
	Cyanobacterial biomass		LC-HRMS/MS (Hybrid Q-Orbitrap)	Column: Accucore aQ UHPLC C18 stationary phase column (100 mm × 2.1 mm, 2.6 μm, Thermo Scientific, USA)<br>Temperature: 60 °C<br>Flow rate: 0.4 mL/min<br>Mobile phases: A—0.025% (v/v) FA in H_2O, B—0.025% (v/v) FA in ACN<br>Injection volume: 10 μL<br>Total run time: 11 min	MC-LR-[Dha7], MC-LR-[Asp3]	NA	NA	
9.	Cyanobacterial biomass, shellfish tissue	Lyophilization–weighing ~100 mg–extraction 75 % ACN with 0.1 % FA–sonication 3 min–vortexing 5 min–centrifugation–decantation–pellet re-extraction (2 times)–filtration, PVDF filter 0.45 μm	HILIC-MS	Column: TOSOH Bioscience column (5 μm TSKGel Amide, 80, 2 × 250 mm)<br>Temperature: 40 °C<br>Flow rate: 450-350 μL/min<br>Mobile phases: A - 50 mM FA with 2 mM ammonium formate in H_2O, B - ACN.<br>Injection volume: 1 μL<br>Total run time: 36 min	17 MCs, NOD-R, ATX-a, hATX, CYN, deoxyCYN, 14 STXs, GNT, LWTX1	0.01 – 0.99 μg g −1	NA	[210]
10.	Water, scum, foam, cyanobacterial biomass	Samples from 2015 for ATX-a: sonication–filtration, filter 0.2 μm–direct injection;<br>other samples–not detailed	LC-MS	Column: Phenomenex Kinetex C18 column (2.1 × 100 mm, 2.6-μm) (MCs, NOD), Agilent Polaris-Ether C18 column (ATX-a, CYN), Cogent diamond hydride column (ATX-a in samples from 2015)<br>Injection volume: 20 μL	11 MCs, NOD-R, CYN, ATX-a	*0.05 – 0.28 ng mL −1	NA	[211]
		Not detailed	UPLC-MS/MS	Column: HSS T3 column (100 mm × 2.1 mm, 1.8-μm, 130 Å pore size, Waters)<br>Temperature: 35 °C<br>Flow rate: 0.45 mL/min<br>Mobile phases: A—0.1% FA in H_2O, B—0.1% FA in ACN<br>Total run time: 12 min		<0.5 ppb	NA	
			LC-MS/SIM	Column: Phenomenex Kinetex C18 column (2.1 × 100 mm, 2.6 μm)		0.05 ng g −1	NA	
		Lysis (freeze–thaw)–filtration, filter 0.7 μm–extraction of the filter with acidic MeOH	LC-MS/MS	Column: Atlantis T3 Column C18 (3 × 150 mm, 3μm)		NA	NA	

				Mobile phases: A—0.1% FA, and 2 mM ammonium formate in H_2O, B—0.1% tetrahydrofuran, and 2 mM ammonium formate in 50:50 (v/v) MeOH:ACN				
11.	Water, cyanobacterial biomass	A) Biomass lyophilization–weighing 10 mg–extraction 75% MeOH: 25% H_2O–vortexing–sonication 15 min–centrifugation–supernatant collection–pellet re-extraction (2 times, last–with n-BuOH instead MeOH:H_2O)–combined supernatant filtration, PVDF filter 0.45 μm–evaporation–reconstitution with 1000 μL MeOH: H_2O (5:95 v/v)<br>B) lyophilization of filter with biomass–filter weighing–extraction 75% MeOH: 25% H_2O–sonication 15 min–centrifugation–supernatant collection–filtration, PVDF filter 0.45 μm–evaporation of 3 mL aliquot–reconstitution with 500 μL MeOH: H_2O (5:95 v/v)–sonication 5 min	LC-MS/MS (for MCs, NOD, CYN, ATX-a)	Column: Atlantis T3 Column, 2.1 mm x 100 mm, 3 μm (Waters, Ireland)<br>Temperature: 30 °C<br>Flow rate: 0.2 mL min−1<br>Mobile phases: A—0.5% FA in ACN, B—0.5% FA in H_2O<br>Injection volume: 20 μL<br>Total run time: 28 min	12 MCs, NOD, CYN, ATX-a, STX, neoSTX	0.1 – 1.0 ng mg −1 dw	0.4 – 2.9 ng mg −1 dw	[207]
		C) water samples filtration–tandem SPE, cartridges (not detailed)				0.8 – 6.5 ng L−1.	NA	
		D) water samples filtration–direct analysis (for STXs)	HILIC-MS (for STXs)	Column: SeQuant ZIC- HILIC (150 mm x 2.1 mm, 3.5 mm, Merck) (for STX, neoSTX)<br>Mobile phases: H_2O, ammonium acetate, ACN and FA (not detailed)		0.1 – 3 μg L−1	NA	
12.	Water, scum	Filtration of 1 mL of water, PVDF filter 0.2 μm (extracellular cyanotoxins);<br>different intracellular toxins extraction protocols tested:<br>1) 600 μL of sample–bead beating;<br>2) 1.5 mL of sample–freeze–thaw;<br>3) 1000 μL of sample–adding reagent A of lysing solution QuickLyseTM (Abraxis, USA)–shaking 2 min–incubation 8 min–adding reagent solution B–shaking 2 min–incubation 8 min	UPLC-MS/MS	Column: ACQUITY BEH C18 UPLC column (2.1 × 100 mm1.7 μm, Waters, UK), VanGuard ACQUITY BEH C18 UPLC pre-column (2.1 × 5 mm, 1.7 μm, Waters, UK)<br>Temperature: 35 °C<br>Flow rate: 0.3 mL/min- 0.45 mL/min<br>Mobile phases: A—0.1% FA in H_2O, B—0.1% FA in ACN<br>Injection volume: 100 μL<br>Total run time: 12 min	17 MCs, NOD, CYN, deoxy-CYN, ATX-a, hATX	NA	0.1 – 0.5 μg L−1	[212]
13.	Water, scum	Filtration–adjusting pH~2with 0.1% FA–lysis (freeze–thaw)–filtration, filter 0.22 μm–online-SPE	online-SPE-LC-HESI-MS/MS	Column: for on-line SPE—Hypersil Gold aQ with C18 column selectivity (20 mm x 2 mm, 12 mm), for LC—Hypersil Gold with C18 column selectivity (100mm x 2.1 mm,1.9 μm)<br>Temperature: 55 °C<br>Flow rate: 525 μL min −1	6 MCs, NOD, CYN, ATX-a	*0.01 – 0.02 μg L−1	**0.03 – 0.07 μg L−1	[213]

				Mobile phases: A—0.1% FA in MeOH, B—0.1% FA in H_2O, C—0.1% FA in ACN<br>Total run time: 7 min				
14.	Water	Water: dual filtration, glass fiber 1.0 µm, nitrocellulose 0.45 µm–SPE<br>(CYN, ATX-a, STX): cartridge Supelclean ENVI-carb (500 mg, 6 mL, Supelco, USA)–evaporation–reconstitution with 1 mL 10:90 (v/v) H_2O:ACN buffered with 5 mM ammonium formate and 3.6 mM FA(pH 3.7)–filtration, filter 0.2 µm;<br>(MCs, NOD): cartridge Oasis HLB (200 mg, 6 mL, Waters, USA)–evaporation–reconstitution with 1 mL 90:10 (v/v) H_2O:ACN buffered with 5 mM ammonium formate and 3.6 mM FA (pH 3.7)–filtration, filter 0.2 µm	LC-MS/MS	Column: ANA, CYN, STX: Poroshell HILIC-Z column (2.1 x 150 mm, 2.7 µm, 120Å, Agilent Technologies, USA)<br>Temperature: 30 °C<br>Flow rate: 0.5 mL min−1<br>Mobile phases: A—5 mM ammonium formate and 3.6 mM FA (pH 3.7) in 5:95 H_2O:ACN (v/v), B—5 mM ammonium formate and 3.6 mM FA (pH 3.7) in H_2O<br>Injection volume: 50 µL	5 MCs, NOD, ATX-a, CYN, STX	0.004 – 0.07 ng mL–1	0.01 – 0.23 ng mL –1	[206]
	Fish tissue	Homogenization of frozen tissue–weighing 1 g–lyophilization–extraction (CYN, ATX-a, STX): 10 mL of 25:75 (v/v) ACN: H_2O; (MCs, NOD): 10 mL of 75:25 (v/v) ACN: 0.1% FA in H_2O–mixing–sonication 30 min–shaking 30 min–centrifugation–supernatant collection–filtration, polypropylene filter 0.45 µm–evaporation–reconstitution with 20 mL H_2O–SPE (same protocols as for water samples)		Column: MCs, NOD: Poroshell SB-C18 RPLC column (2.1 × 100 mm, 2.7 m, 120Å, Agilent Technologies, USA)<br>Temperature: 60 °C<br>Flow rate: 0.5 mL min−1<br>Mobile phases: A—5 mM ammonium formate and 3.6 mM FA (pH 3.7) in H_2O, B—5 mM ammonium formate and 3.6 mM FA (pH 3.7) in 5:95 H_2O:ACN (v/v),<br>Injection volume: 50 µL		0.02 – 0.08 ng mL –1	0.07 – 0.28 ng mL –1	
15.	Water	Not detailed	online-SPE–UHPLC-MS/MS	Column: for on-line SPE—Hypersil Gold aQ C18 column (20 mm × 2.1 mm, 12 µm); for LC—Thermo Hypersil Gold C18 column (100 mm × 2.1 mm, 1.9 µm, Thermo Fisher Scientific, USA) equipped with a 0.2 µm column pre-filter.<br>Temperature: 55 °C<br>Mobile phases: A—0.1% FA in H_2O, B—0.1% FA in MeOH, C—0.1% FA in ACN<br>Flow rate: 525 µL min –1<br>Total run time: 8 min	12 MCs, ATX-a, hATX, CYN, cyanopeptolin A, anabaenopeptins A and B (AptA, AptB)	0.1 – 10 µg kg –1	0.3 – 33 µg kg –1	[214]
	Fish tissue	Homogenization–weighing 500 mg–extraction MeOH–vortexing–sonication 15 min–centrifugation–supernatant collection –re-extraction (2 times)–combined supernatant evaporation–reconstitution with H_2O–vortexing–sonication 5 min–filtration, GHP filter 0.2 µm						

16.	Water	Water: filtration–pH 11 adjusting–tandem SPE, cartridges Supel-Select HLB (200 mg, 6 mL, Supelco) and Supelclean ENVICarb (250 mg, 3 mL, Supelco)–evaporation–reconstitution with 150 μL of 5% (v/v) MeOH (extracellular cyanotoxins); filter: extraction with 9 mL 75% (v/v) MeOH–centrifugation–evaporation of 3 mL aliquot–reconstitution with 500 μL of 5% (v/v) MeOH (intracellular cyanotoxins)	LC-MS/MS	NA	12 MCs, NOD, CYN, ATX-a	0.001 – 0.007 μg L–1	0.003 – 0.02 μg L–1	[179]
	Fish tissue	Lyophilizationn–weighing 0.2 g–extraction 5 mL 80% MeOH containing 0.5% HCOOH–stirring 15 min–sonication 30 min–centrifugation–supernatant extraction 1 mL of hexane (3 times)–hexane phase discarded–dilution of bottom layer with 100 mL of H_2O with 0.3% FA–SPE Supel-Select HLB (200 mg, 6 mL, Supelco)–evaporation–reconstitution with 200 μL of 5% (v/v) MeOH (for MCs)						
17.	Water	Filtration of water sample, Sterivex filter–filtrate collection (extracellular toxins)–lysis (freeze–thaw of the filter)–rinsing filter with MeOH:H_2O (1:1)–filtrate collection in a vial (intracellular toxins)	RP-UPLC-MS/MS	Column: ACQUITY BEH C18 UPLC column (2.1 × 100 mm, 1.7 μm) fitted with a pre-column VanGuard ACQUITY BEH C18 UPLC, (2.1 × 5 mm, 1.7 μm, Waters, UK)<br>Temperature: 35 °C<br>Flow rate: 0.3 mL/min – 0.45 mL/min<br>Mobile phases: A—0.1 % FA in H_2O, B—0.1 % FA in ACN<br>Injection volume: 100 μL<br>Total run time: 12 min	17 MCs, NOD, ATX-a, hATX, CYN	NA	0.1 μg L−1	[167]
			UPLC-HILIC-MS/MS	Column: HILIC column (not indicated)<br>Flow rate: 0.4 mL/min – 0.8 mL/min<br>Mobile phases: A1—H_2O/FA/NH4OH (500:0.075:0.3 v/v/v), B1—ACN/H_2O/FA (700:300:0.1 v/v/v); A2—H_2O/FA (200:1 v/v), B2—MeOH<br>Total run time: ~10.5 min	STX, dcSTX, dcneoSTX	NA		
18.	Water	Filtration, filter 0.45 μm–spiking of 80.0 mL freshwater with 1% NaCl–SPE, cartridge ENVI-Carb (PGC, 250 mg, 3 mL, Supelco, USA)	online-SPE-HILIC-MS/MS	Column: for on-line SPE—A zwitterionic HILIC (ZIC-HILIC) guard column (2.1 mm × 20 mm, 5.0 μm, Merk), for LC—Poroshell 120 HILIC-Z column (2.1 mm × 50 mm, 1.9 μm, Agilent)<br>Temperature: 30 °C<br>Flow rate: 0.4 mL/min	ATX-a, CYN, 13 STXs	0.7 – 4.1 ng L–1	1.8 – 8.1 ng L–1	[215]

				Mobile phases: A—2 mM ammonium formate and 0.03% FA in H_2O, B—2 mM ammonium formate and 0.03% FA in 90% aqueous ACN<br>Injection volume: 10 μL<br>Total run time: 21 min				
			HILIC-MS/MS	Column: 3 columns were tested: HILIC-Z, HILIC-Si, HILIC-Amide; HILIC-Si – HILIC Plus RRHD column (3.0 mm × 100 mm, 1.8 μm, Agilent)<br>Temperature: 30℃<br>Flow rate: 0.4 mL/min<br>Mobile phases: A—5 mM ammonium formate and 0.2% FA in H_2O, B—5 mM ammonium formate and 0.2% FA in ACN<br>Injection volume: 10 μL<br>Total run time: 21 min		1.1 – 54.5 ng L–1	2.5 – 130.4 ng L–1	
19.	Water	Aliquot (A)–filtration, filter 0.45 μm, aliquot (B)–lysis (freeze–thaw);<br>CYN, ATX-a, hATX: 1 mL from aliquot (A), 1 mL from aliquot (B)–filtration, filter 0.22 μm;<br>MCs, NOD, AptA, AptB: samples from aliquots (A) and (B)–SPE, cartridge, Oasis HLB (200 mg, 6 mL, Waters, USA)–evaporation–reconstitution with 1 mL of MeOH:ACN:H_2O (10:10:80, v/v/v) in 0.1% FA	LC-MS/MS	Column: Agilent Poroshell 120 EC18 reverse phase column (100 × 4.6 mm, 2.7 μm, Agilent, USA)<br>Mobile phases: A—0.1% FA in H_2O, B—0.1% FA in 50/50 v/v ACN/MeOH<br>Injection volume: 20 μL<br>Total run time: 27 min	12 MCs, CYN, ATX-a, hATX, NOD, anabaenopeptin A (AptA) and anabaenopeptin B (AptB)	*0.02 – 33 ng L–1	**0.06 – 100 ng L–1	[205]
20.	Water	Filtration, filter 0.22 μm–tandem SPE, cartridges Strata-X cartridges (Phenomenex, USA) then MCX different sorbent mass (60 mg and 150 mg)–evaporation–reconstitution with 250 μL of 60% ACN with 0.1% of FA–filtration, PTFE filter 0.2 μm	HILIC-MS/MS	Column: SeQuant ZIC-HILIC (2.1 × 250 mm, 3.5 μm, EMD Millipore, USA)<br>Mobile phases: A—0.3% (v/v) FA in H_2O, B—0.3% (v/v) FA in ACN<br>Injection volume: 20 μL<br>Total run time: 35 min	MC-LR, MC-RR, NOD, CYN, ATX-a, BMAA, 2,4-DAB, AEG	0.0012 – 0.03 μg L–1	0.004 – 0.1 μg L–1	[166]
21.	Water	Filtration, filter 1.2 μm–refiltration, filter 0.22 μm–direct injection (extracellular cyanotoxins)<br>1.2 μm filter: lyophilization–bead beating extraction with MeOH:H_2O (75:25, v/v)–centrifugation–liquid separation–dilution–filtration, filter 0.22 μm (intracellular cyanotoxins)	UHPLC-HRMS	Column: RP core-shell Kinetex® biphenyl column (100 x 2.1 mm, 2.6 mm, 100 Å, Phenomenex, the Netherlands)<br>Temperature: 45 °C<br>Flow rate: 0.3 mL/min<br>Mobile phases: A—0.05% (v/v) acetic acid in H_2O, B—MeOH<br>Injection volume: 20 μL<br>Total run time: 20 min	6MCs, CYN, ATX-a, hATX	*3 – 45 ng L–1	**8 – 45 ng L–1	[142]

22.	Water	Filtration, filter 0.7 μm–pH 11 adjusting–tandem SPE, cartridges Supel-Select HLB (200 mg, 6 mL, Supelco, USA) and Supelclean ENVI-Carb (250 mg, 3 mL, Supelco, USA)–evaporation–reconstitution with 400 μL MeOH: H_2O (5:95, v/v); filter: extraction with 75% MeOH:25% H_2O–centrifugation–evaporation–reconstitution with MeOH:H_2O (5:95, v/v) (intracellular cyanotoxins)	LC-MS/MS	Column: Atlantis T3 (2.1 mm x 100 mm, 3 μm, Waters, Ireland) Temperature: 30 °C Flow rate: 0.2 mL/min Mobile phases: A—0.5% FA in ACN, B—0.5% FA in H_2O Injection volume: 20 μL Total run time: 18 min	12 MCs, NOD, CYN, ATX-a, and 10 cyanopeptides (3 APs, 4 MGs, 2 AERs and aeruginosamide)	0.001 – 0.007 μg L–1	0.003 – 0.02 μg L–1	[216]
23.	Water	Ultrasonication 30 min–centrifugation–supernatant collection–tandem SPE, cartridges Oasis HLB (500 mg, 6 mL, Waters, USA) and Supelclean ENVI-Carb (500 mg, 6 mL, Supelco, USA)–evaporation–reconstitution 500 μL ACN:H_2O (10:90, v/v) (total cyanotoxins)	UHPLC-HRMS	Column: C18 UHPLC column Hibar® (2.1 mm x 150 mm, 2 μm) Flow rate: 0.3 mL/min Mobile phases: A—0.1% FA in H_2O, B—0.1% FA in ACN Injection volume: 20 μL Total run time: 10 min	7 MCs, CYN, ATX-a, NOD	4 – 150 pg L–1	12 – 450 pg L–1	[143]
24.	Water	Filtration–pH 11 adjusting–tandem SPE, cartridges Oasis HLB (200 mg, 6 mL, 25–35 μm, Waters, USA) and HyperSep Hypercarb PGC (200 mg, 3 mL, 30–40 μm, Thermo Scientific, UK)–evaporation–reconstitution with 400 μL MeOH:H_2O (5:95, v/v)–sonication	LC-MS/MS	Column: Atlantis T3 (2.1 mm × 100 mm, 3 μm); Atlantis dC18 (2.1 mm × 100 mm, 3 μm, Waters, Ireland); Zorbax eclipse XDB-C18 (4.6 mm × 50 mm, 1.8 μm); Poroshell120 EC-C18 (2.1 mm × 100 mm, 2.7 μm, Agilent, USA) Temperature: 30 °C Flow rate: 0.2 mL/min Mobile phases: A—0.5% FA in ACN, B—0.5% FA in H_2O Injection volume: 20 μL Total run time: 28 min	12 MCs NOD, CYN, ATX-a	1 – 10 ng L –1	NA	[217]
25.	Water	Concentration of 50 L water using hemoflow cartridge (60 Da exclusion size)–freezing of 40 mL aliquots–sonication (3 times)–centrifugation–separation 10 mL aliquots–evaporation–reconstitution with 1 mL MeOH–centrifugation–filtration, filter 0.45-μm–evaporation–reconstitution with PBS-BT	UPLC-MS/MS	Column: Aquity HSS T3 column (100 mm x 2.1 mm, 1.8 μm, Waters) Temperature: 35 °C Flow rate: 0.45 mL/min Mobile phases: A—0.05% FA in H_2O, B—0.05% FA in ACN Injection volume: 5 μL Total run time: 12.5 min	3 MCs, NOD, CYN, ATX-a, hATX, dihydro derivatives of ATX-a (H_2-homoATX-a, H_2-ATX-a)	0.0038 – 0.0078 μg L–1	0.01 – 0.02 μg L–1	[218]
26.	Water	Not detailed	LC-QTOF HRMS	Column: Accucore C-18 (100 mm x 2.1 mm, 2.6 μm, Superchrom, Italy) Temperature: 30 °C Flow rate: 0.3 mL/min Mobile phases: A—10 mM FA in H_2O, B—10 mM FA in ACN Injection volume: 20 μL Total run time: 25 min	9 MCs, NOD, CYN, ATX-a and standardless cyanotoxins from database	NA	NA	[219]

27.	Water	Collected drinking water mixed with sodium thiosulfate to a concentration of 100 mg/L–3 mL of water–centrifugation–separation 0.9 mL aliquots–mixed with 0.1 mL internal standard in ACN (extracellular cyanotoxins); lysis of 3 mL of water (freeze–thaw, 3 times)–sonication (3 times)–centrifugation–separation 0.9 mL aliquots–mixed with 0.1 mL internal standard in ACN (total cyanotoxins)	LC-MS/MS	Column: Hypersil Gold C18 Selectivity LC column (2.1 × 100 mm, 3 μm) with a Hypersil Gold drop-in guard column (2.1 × 10 mm, 3 μm, Thermo Scientific, TN)<br>Temperature: 30°C<br>Flow rate: 500 μL/min<br>Mobile phases: A—0.1% FA in H_2O and ACN (9 + 1, v/v), B—0.1% FA in MeOH and H_2O (9 + 1, v/v)<br>Injection volume: 25 μL<br>Total run time: ~12 min	8 MCs, NOD, CYN, ATX-a	0.03 – 0.09 μg L–1	0.1 – 30 μg L–1	[220]
28.	Water	Filtration–filtrate lyophilization–reconstitution with MeOH (extracellular cyanotoxins)<br>lyophilization of the filter–extraction 100 % MeOH–sonication 20 min–centrifugation–filtrate collection–pellet re-extraction (2 times) filtration, PTFE filter 0.2 μm–evaporation–reconstitution with 1 mL H_2O:MeOH (90:10, %) in a separate vial–pellet re-extraction MeOH:H_2O (75:25, %) sonication 15 min–centrifugation–filtrate collection)–filtration–evaporation–reconstitution with 1 mL H_2O:MeOH (90:10, %) (intracellular cyanotoxins)	LC-MS/MS	Column: Atlantis dC18 column (100 x 2.1 mm, 3 μm), security guard cartridge Atlantis dC18 (20 x 2.1mm, 3 μm)<br>Temperature: 35 °C<br>Flow rate: 0.3 mL min –1<br>Mobile phases: A—0.1% FA in H_2O, B—0.1% FA in ACN<br>Injection volume: 20-μL<br>Total run time: 29 min	6 MCs, NOD, CYN, ATX-a	0.04 – 0.8 mg L–1	0.1 – 2.3 mg L–1	[221]
29.	Water	Direct injection method: 2 mL of water–filtration, filter 0.22 μm (extracellular cyanotoxins);<br>5 mL of water–lysis (freeze–thaw)–sonication 10 min–filtration, filter 0.22 μm (total cyanotoxins);	LC-MS/MS	Column: Agilent Poroshell 120 EC-C18 (3.0 × 100 mm, 2.7 μm), Agilent ZORBAX SB-C18 (2.1 × 150mm, 3.5 μm), Agilent RRHD Eclipse Plus C18 (2.1 × 100 mm, 1.8 μm)<br>Mobile phases: A—0.1% FA in H_2O, B—0.1% FA in 50:50 (v/v) MeOH:ACN<br>Injection volume: 20 μL<br>Total run time: 27 min	CYN, ATX-a, hATX	*15 – 70 ng L–1	**50 – 240 ng L–1	[190]
		SPE method: 500 mL of water–SPE, different cartridges tested Chromabond® HR-X (500 mg, 6 mL), Oasis HLB (200 mg, 6 mL), Supelclean Coconut Charcoal (2000 mg, 6 mL)–evaporation–reconstitution with 1mL MeOH:ACN:H_2O (10:10:80, v/v/v) in 0.1% FA (LOD/LOQ for CYN not determined (low recovery)				*0.6 – 1.3 ng L–1	**2 – 4 ng L–1	
30.	Water	Filtration, filter 0.45 μm–filtrate freezing–acidifying with FA to 0.1% FA in the volume–evaporation;<br>filter with biomass–extraction with ACN:H_2O:FA (80:19.9:0.1) (3 times)–supernatant collection–evaporation–reconstitution of filtrates with 500 μL 75% ACN (for PSPs), 50% MeOH (for MCs), 0.1% FA (for CYNs, ATX-a)	LC-MS/MS	Column: Purospher STAR RP-18 end-capped column (30 x 4 mm, 3 μm, Merck, Germany)<br>Temperature: 30 °C<br>Flow rate: 0.5 mL/min<br>Mobile phases: A—0.5% FA in H_2O, B—0.5% FA in ACN<br>Injection volume: 10 μL<br>Total run time: 11 min	8 MCs, CYN, deoxy-CYN, ATX-a, 12 STXs	NA	0.04 – 0.5 μg L –1	[83]

				Column: Waters Atlantis C18 column (2.1 x 150 mm, 5 μm)<br>Temperature: 30 °C<br>Flow rate: 0.25 mL min –1<br>Mobile phases: A—0.1% FA in H_2O, B—0.1% FA in MeOH, linear gradient from 1% to 25% MeOH in H_2O<br>Injection volume: 10 μL<br>Total run time: 5 min		NA	0.01 – 0.02 μg L –1	
				Column: TSK gel Amide-80 (2 x 250 mm, 5 μm, Tosoh, Germany)<br>Temperature: 30 °C<br>Flow rate: 0.2 mL min –1<br>Mobile phases: A—2.0 mM ammonium formate and 3.6 mM FA (pH 3.5) in H_2O, B—2.0 mM ammonium formate and 3.6 mM FA (pH 3.5) in ACN-H_2O (95:5)<br>Injection volume: 10 μL<br>Total run time: ~33 min		NA	0.1 – 2 μg L–1	
31.	Water	Filtration–freeze–thaw of filter–extraction 75% MeOH–sonication	LC-MS/MS	Column: Thermo Hypersil Gold (100 × 3 mm, 3 μm)<br>Mobile phases: A—0.05% FA in H_2O, B—0.05% FA in ACN<br>Flow rate: 0.2 mL/min;<br>Injected volume: 25 μL	5 MCs, ATX-a	NA	NA	[222]
32.	Water	freezing of water samples–filtration of 200μL, 0.2 μm PTFE filter	UPLC-MS/MS	Column: Acquity UHPLC HSS T3 column (100 mm × 2.1 mm, 1.8 μm, 130 Å, Waters)<br>Temperature: 35 °C<br>Flow rate: 0.45 mL/min<br>Mobile phases: A—0.1% FA in H_2O, B—0.1% FA in ACN<br>Total run time: 12 min	11 MCs, ATX-a, CYN, deoxy-CYN, AptA, AptB, euglenophycin	0.5 μg L–1	NA	[223]
		SPATT: DIAION HP20 resin was used<br>Extraction protocol: not detailed	LC-MS/SIM	Column: Phenomenex Kinetix C18 (100 mm x 2.1mm)	5 MCs, NOD-R, ATX-a, hATX, CYN	<0.5 ng g –1	NA	
33	Water	3 freeze–thawing cycles–filtration, filter 0.2 μm, GHP filters	Online-SPE-UHPLC-HRMS	Column: for on-line SPE—Hypersil Gold aQ (20 mm x 2 mm, 12 μm, 175 Å), for LC—Hypersil Gold with C18 column selectivity (100mm x 2.1 mm,1.9 μm, 175 Å)<br>Temperature: 55 °C<br>Flow rate: 550 μL min –1	12 MCs, CYN ATXs, AptA, AptB	*8 – 53 ng L–1	**27 – 176 ng L–1	[224]

				Mobile phases: A—0.1% FA in H_2O, B—0.1% FA in ACN<br>Total run time: 8 min				
34.	Soil	Soil air drying 48–grinding–sieving <2 mm mesh–homogenization–weighing 1 g–extraction (different MeOH-based solvents and volumes used; MeOH with 200 mM ammonium acetate–the best result)–blending–sonication 15 min–centrifugation–supernatant collection–soil re-extraction (different number of cycles used)–combined supernatant evaporation–reconstitution with 2 mL H_2O–vortexing–filtration, GHP filter	Online-SPE-UHPLC-MS/MS	Column: for on-line SPE—Thermo Hypersil Gold aQ column (C18, 20 mm × 2 mm, 12 µm); for LC—Thermo Hypersil Gold C18 (100 mm × 2.1 mm, 1.9 µm)<br>Temperature: 55 °C<br>Flow rate: 500 µL min−1	10 MCs, NOD-R, ATX-a, hATX, CYN, AptA, AptB, cyanopeptolin A	0.001 – 0.3 ng g −1	0.003 – 0.9 ng g −1	[225]
35.	Plant leaves	Weighing 1 g–lyophilization–80 % MeOH extraction–homogenization–sonication 15 min–stirring 15 min–centrifugation–supernatant collection–pH adjusting (different pH tested)–tandem SPE, cartridges C18 Bakerbond® (500 mg, 6 mL, Dicsa, Spain) and BOND ELUT® Carbon cartridge (Agilent, the Netherlands)–extract evaporation–reconstitution with 1 mL 20% MeOH	UPLC-MS/MS	Column: XSelect HSS T3 column (100 x 2.1 mm, 2.5 µm)<br>Flow rate: 0.45 mL min −1<br>Mobile phases: A—0.1% FA in H_2O, B—0.1% FA in ACN<br>Injection volume: 5 µL<br>Total run time: 9.2 min	3 MCs, CYN	0.06 – 0.42 ng g −1 fw	0.16 – 0.91 ng g −1 fw	[226]
36.	Plant leaves	MCs, CYN: lyophilization–weighing 0.05 g–extraction 80% MeOH–homogenization–sonication–centrifugation–supernatant collection–pH 11 adjusting–tandem SPE, cartridges Bakerbond® C18 (Dicsa, Spain) and Bond Elut Carbon PGC (Agilent, USA)–evaporation–reconstitution with 20% MeOH;<br>samples spiked with CYN: lyophilization–weighing 0.05 g–extraction 10% acetic acid–homogenization–stirring–sonication–centrifugation–supernatant collection–pH 11 adjusting–SPE, cartridge Bond Elut Carbon PGC (Agilent, USA)–evaporation–reconstitution with H_2O	UPLC-MS/MS	Column: XSelect HSS T3 column (100 × 2.1 mm, 2.5 µm)<br>Flow rate: 0.45 mL min −1<br>Mobile phases: A—H_2O, B—ACN<br>Injection volume: 5 µL<br>Total run time: 10 min	3 MCs, CYN	NA	NA	[227]

Abbreviations:

MCs—microcystins, MC-—microcystin-, NOD—nodularin, NOD-R—nodularin-R, GNT—guanitoxin, AptA—anabaenopeptin A, AptB—anabaenopeptin B, LWTX1—lyngbyatoxin 1, ATXs—anatoxins, ATX-a—anatoxin-a, hATX—homoanatoxin, CYNs—cylindrospermopsins, CYN—cylindrospermopsin, deoxy-CYN—deoxy-cylindrospermopsin, AETX—aetokthonotoxin, STX—saxitoxin, STXs—saxitoxin analogues, neoSTX—neosaxitoxin, dcneoSTX—decarbamoylneosaxitoxin, dcSTX—decarbamoylsaxitoxin, BMAA—ß-methylamino-L-alanine, 2,4-DAB—2,4-diaminobutyric acid, AEG—aminoethyl glycine, fw—fresh weight, dw—dry weight, HILIC—hydrophilic interaction liquid chromatography, HPLC-MS—high-performance liquid chromatography mass spectrometry, LC-MS—liquid chromatography mass spectrometry, LC-MS/MS—liquid chromatography coupled with tandem mass spectrometry, UHPLC-MS/MS—ultra-high performance liquid chromatography coupled with tandem mass spectrometry, RP-UPLC-MS/MS—reverse-phase ultra-performance liquid chromatography coupled with tandem mass spectrometry, HRMS—high-resolution mass spectrometry, ESI—electrospray ionization, HESI—heated electrospray ionization, SIM—selected ion monitoring, SPE—solid-phase extraction, ACN—acetonitrile, MeOH—methanol, FA—formic acid, DCM—dichloromethane, PBS—phosphate-buffered saline solution, SPATT—solid-phase adsorption toxin tracking (passive sampler), GHP—hydrophilic polypropylene, PGC—porous graphitic carbon, PVDF—polyvinylidene difluoride

a - Manufacturer information is provided only when reported in the original publication.
* - Method detection limit (MDL)
* * - Method quantification limit (MQL)
